# IBR5 Modulates Temperature-Dependent, R Protein CHS3-Mediated Defense Responses in *Arabidopsis*

**DOI:** 10.1371/journal.pgen.1005584

**Published:** 2015-10-09

**Authors:** Jingyan Liu, Haibian Yang, Fei Bao, Kevin Ao, Xiaoyan Zhang, Yuelin Zhang, Shuhua Yang

**Affiliations:** 1 State Key Laboratory of Plant Physiology and Biochemistry, College of Biological Sciences, National Plant Gene Research Center, China Agricultural University, Beijing, China; 2 Department of Botany, University of British Columbia, Vancouver, British Columbia, Canada; University of California Davis, UNITED STATES

## Abstract

Plant responses to low temperature are tightly associated with defense responses. We previously characterized the chilling-sensitive mutant *chs3-1* resulting from the activation of the Toll and interleukin 1 receptor-nucleotide binding-leucine-rich repeat (TIR-NB-LRR)-type resistance (R) protein harboring a C-terminal LIM (Lin-11, Isl-1 and Mec-3 domains) domain. Here we report the identification of a suppressor of *chs3*, *ibr5-7* (*indole-3-butyric acid response 5*), which largely suppresses chilling-activated defense responses. *IBR5* encodes a putative dual-specificity protein phosphatase. The accumulation of CHS3 protein at chilling temperatures is inhibited by the *IBR5* mutation. Moreover, *chs3*-conferred defense phenotypes were synergistically suppressed by mutations in *HSP90* and *IBR5*. Further analysis showed that IBR5, with holdase activity, physically associates with CHS3, HSP90 and SGT1b (Suppressor of the G2 allele of *skp1*) to form a complex that protects CHS3. In addition to the positive role of IBR5 in regulating CHS3, *IBR5* is also involved in defense responses mediated by *R* genes, including *SNC1* (*Suppressor of npr1-1*, *Constitutive 1*), *RPS4* (*Resistance to P*. *syringae 4*) and *RPM1* (*Resistance to Pseudomonas syringae pv*. *maculicola 1*). Thus, the results of the present study reveal a role for IBR5 in the regulation of multiple R protein-mediated defense responses.

## Author Summary

Resistance (*R*) genes play central roles in recognizing pathogens and triggering plant defense responses. *CHS3* encodes a TIR-NB-LRR-type R protein harboring a C-terminal LIM domain. A point mutation in CHS3 activates the defense response under chilling stress. Here we identified and characterized *ibr5-7*, a mutant that suppresses the chilling-induced defense responses of *chs3-1*. We observed that the enhanced defense responses and cell death in the *chs3-1* mutant are synergistically dependent on *IBR5* and *HSP90*. IBR5 physically interacts with CHS3, forming a complex with SGT1b/ HSP90. Moreover, IBR5 is also involved in the *R*-gene resistance mediated by *SNC1*, *RPS4* and *RPM1*. Thus, IBR5 plays key roles in regulating defense responses mediated by multiple R proteins.

## Introduction

Plant growth and development are continuously affected by various environmental stresses, including low temperature and pathogen infection. Emerging evidence has shown that the defense responses of plants are regulated by temperatures [1]. Temperature modulates the defense responses induced by certain types of resistance (R)/R-like proteins, including Toll/Interleukin–1 receptor (TIR)- nucleotide-binding (NB)- leucine-rich repeat (LRR) proteins [N (Resistance to tobacco mosaic virus) in tobacco; RPP1 (Recognition of *Peronospora parasitica* 1)-like, RPP4, RPS4 (Resistance to *P*. *syringae* 4) and SNC1 (Suppressor of *npr1-1*, Constitutive 1) in *Arabidopsis*], CC-NB-LRR proteins [Rx in tomato; RPM1 (Resistance to *Pseudomonas syringae pv*. maculicola 1) and RPS2 in *Arabidopsis*], LRR-TM (transmembrane-domain) proteins (Cf–4 and Cf–9 in tomato), TM-CC proteins [RPW8 (Resistance to Powdery Mildew 8) in *Arabidopsis*] [2–7], and the TIR-NB protein CHS1 (chilling sensitive 1) in *Arabidopsis* [8]. A recent study showed that low temperatures (10°C to 23°C) elevate R protein-mediated effector-triggered immunity (ETI), and higher temperatures (23°C to 32°C) lead to a shift in pattern-triggered immunity (PTI) signaling in plants [9]. These studies suggest that temperature largely affects the function of R proteins.

Recent studies have revealed that a number of components regulate the activities of R proteins, which in turn finely tune defense signaling. Chaperone and co-chaperone proteins, such as the HSP90-SGT1b (Suppressor of the G2 allele of *skp1*)-RAR1 (Required for MLA12 resistance 1) complex, involve in multiple R protein-mediated defense pathways [10]. Earlier reports have indicated that these complexes are required for the correct folding and/or stability of R proteins [11–13]. However, several recent studies have suggested that both SGT1b and HSP90 play positive roles in the degradation of R proteins, including RPM1, RPS2 and SNC1, by the SCF complex [14–16]. The dual functions of R protein regulators ensure that the plant immunity system rapidly and properly responds to pathogen invasion. In addition to these chaperones, many other regulators are also involved in R protein regulation. A series of regulators of SNC1 were identified by screening suppressors and enhancers of *snc1-1*, have been shown to regulate the chromatin, transcription and protein levels of SNC1. These proteins include E1 and E4 ligases, U-box proteins, acetyltransferases, RNA binding proteins, nuclear pore complex components [13,17–22]. These results suggest that SNC1 and/or other R proteins are regulated by multiple biological processes including nucleo-cytoplasmic trafficking, transcriptional reprogramming, RNA processing and protein modification.

Previous studies have shown that low temperature activates defense responses in plants harboring mutations in *R/R-like* genes, including *CHS1*, *CHS2/RPP4* and *CHS3* [7,8,23]. Arabidopsis *CHS3* encodes a TIR-NB-LRR-type R protein harboring a C-terminal LIM domain [23,24]. The *chs3-1* mutant exhibits chilling-sensitive phenotypes, including small stature and increased disease resistance. The SGT1b and RAR1 proteins are required for R protein stability [25–27]. The *chs3* chilling-sensitive phenotypes are suppressed in *sgt1b* and *rar1* mutants [23]. However, the molecular regulatory mechanism of the temperature-dependent defense responses through CHS3 remains elusive.

In the present study, we identified *ibr5-7* as a suppressor of the chilling-sensitive phenotypes of *chs3-1*. *IBR5* (*Indole-3-Butyric Acid Response 5*) encodes a dual-specificity MAPK phosphatase, which acts as a positive regulator of plant responses to auxin and ABA [28]. IBR5 physically interacts with MPK12, and activated MPK12 is dephosphorylated and inactivated by IBR5, thereby negatively regulating auxin signaling [29]. Here, we observed that *ibr5* suppresses the chilling-sensitive phenotypes of *chs3-1* independently of MPK12. Biochemical data showed that IBR5 complexes with CHS3 and HSP90-SGT1b to to stabilize CHS3. Moreover, IBR5 is involved in the *R*-gene mediated resistance specified by *SNC1*, *RPS4* and *RPM1*. Thus, IBR5 plays an important role in regulating different R protein-mediated defense responses.

## Results

### Identification of the suppressor of *chs3-1*, *suc5*

**Fig 1:**
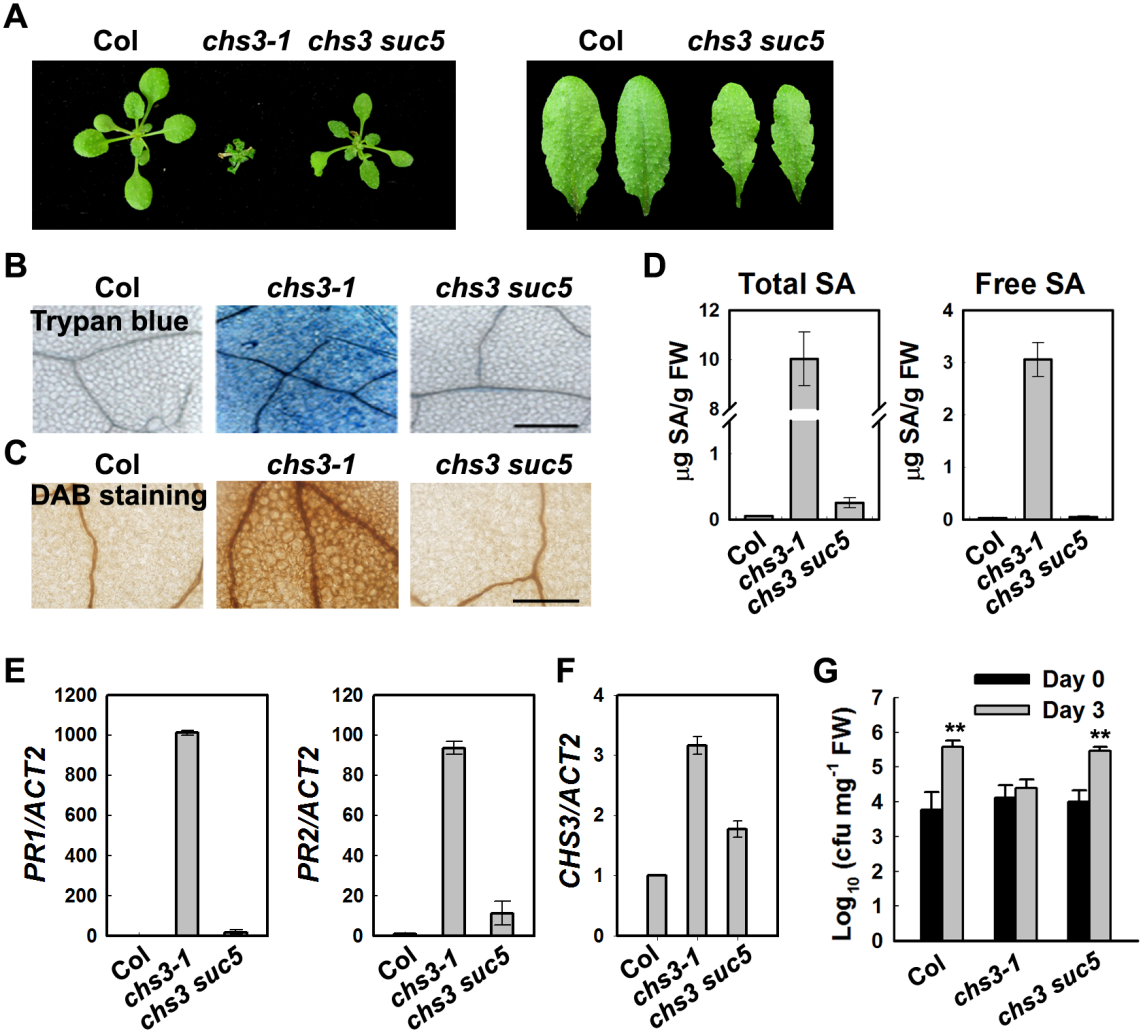
Identification of a suppressor of *chs3-1*, *suc5*. (A) Morphology of wild-type (Col), *chs3-1* and *chs3 suc5* plants grown at 16°C. The photographs show 4-week-old plants grown in soil (left panel). The rosette leaves of *chs3 suc5* have increased leaf serration compared with Col (right panel). (B, C) Trypan blue and DAB staining of leaves from 3-week-old wild type, *chs3-1* and *chs3 suc5* plants grown at 16°C. Bars: 200 μm. (D) Levels of total and free SA in 4-week-old, wild-type, *chs3-1* and *chs3 suc5* plants grown at 16°C. (E, F) Expression of *PR1*, *PR2* (E) and *CHS3* (F) genes in 3-week-old wild-type, *chs3-1* and *chs3 suc5* plants grown at 16°C. The values were normalized to the expression of *ACTIN2*. The error bars represent the SD of three replicates. Similar results were obtained in three independent experiments. (G) Growth of *P*.*s*.*t*. DC3000 on wild-type, *chs3-1*, and *chs3 suc5* plants. Three-week-old plants were dipped with *P*.*s*.*t*. DC3000 (OD_600_ = 0.05). Bacterial growth was monitored as described in the Materials and Methods. The error bars represent the SD of three replicates, and the asterisks indicate significant differences compared with wild-type Col (**P < 0.01, *t*-test).

The *chs3-1* plants are dwarfed and have small, curly leaves. The defense responses in *chs3-1* are constitutively active at 16°C, but this phenotype is alleviated at higher temperatures (22°C) [23]. To understand the molecular mechanism underlying the temperature-dependent cell death in the *chs3-1* mutant, we performed a genetic screen to identify suppressors of *chs3-1* (*suc*). The *chs3-1* seeds were mutagenized with ethyl methylsulfonate (EMS), and the M2 population was screened for mutants with wild-type morphology at 16°C. Among the suppressors screened, most of the variations were second-site, loss-of-function mutations in *CHS3*, thereby rescuing the *chs3* chilling-sensitive phenotype. One suppressor harbored a mutation in *RAR1*, and two suppressors harbored mutations in *SGT1b*; these genes are important regulators required for *CHS3* function [23]. These results indicate that the genetic screen was effective. Here, we characterized *suc5* as a new suppressor of *chs3-1*. The *chs3 suc5* mutant plants largely resembled wild-type plants when grown at 16°C, except these plants exhibited a slightly smaller stature compared with the wild type and had serrated true leaves (Fig 1A).

### *suc5* suppresses cell death and defense responses of *chs3-1* upon chilling stress

Previous studies have indicated that extensive cell death and strong defense responses occur in *chs3-1* mutants grown at 16°C [23]. To determine whether the *suc5* mutation affects these cell death-related phenotypes, *chs3-1* plants were grown at 16°C, followed by staining with trypan blue and 3, 3’-diaminobenzidine (DAB). The *suc5* mutation dramatically reduced the extensive cell death observed in *chs3-1* mutants grown at 16°C (Fig 1B). Furthermore, the accumulation of hydrogen peroxide (H_2_O_2_) in *chs3 suc5* plants grown at 16°C was dramatically reduced compared with *chs3-1* (Fig 1C).

The *chs3-1* mutant also accumulates high levels of salicylic acid (SA) [23]. To determine whether *suc5* inhibits SA accumulation in *chs3-1* at 16°C, the endogenous SA level in *chs3 suc5* plants was measured. Both the free and total SA levels were dramatically reduced in *chs3 suc5* plants grown at 16°C compared with the *chs3* mutant (Fig 1D). Because *PR* genes were highly expressed in *chs3-1*, we further examined the expression of the *PR* genes in *chs3 suc5* plants grown at 16°C. Quantitative real-time PCR (qRT-PCR) analysis showed that the expression of *PR1*, *PR2* and *CHS3* was significantly reduced in *chs3 suc5* plants grown at 16°C (Fig 1E and 1F).

Compared with wild-type plants, *chs3-1* plants grown at 16°C exhibit enhanced resistance to a virulent pathogenic strain of *Pseudomonas syringae* pv tomato (*P*.*s*.*t*.), DC3000 [23]. To investigate the role of *SUC5* in the *chs3*-mediated basal defense response, the response of *chs3 suc5* seedlings to *P*.*s*.*t*. DC3000 was analyzed. The *suc5* mutation fully suppressed the *chs3*-conferred constitutive resistance to *P*.*s*.*t*. DC3000, resulting in wild-type-like susceptibility (Fig 1G). Taken together, these results demonstrated that, under chilling stress, the *suc5* mutation largely suppresses all known autoimmune phenotypes of *chs3*.

### The *SUC5* gene is *IBR5*

**Fig 2:**
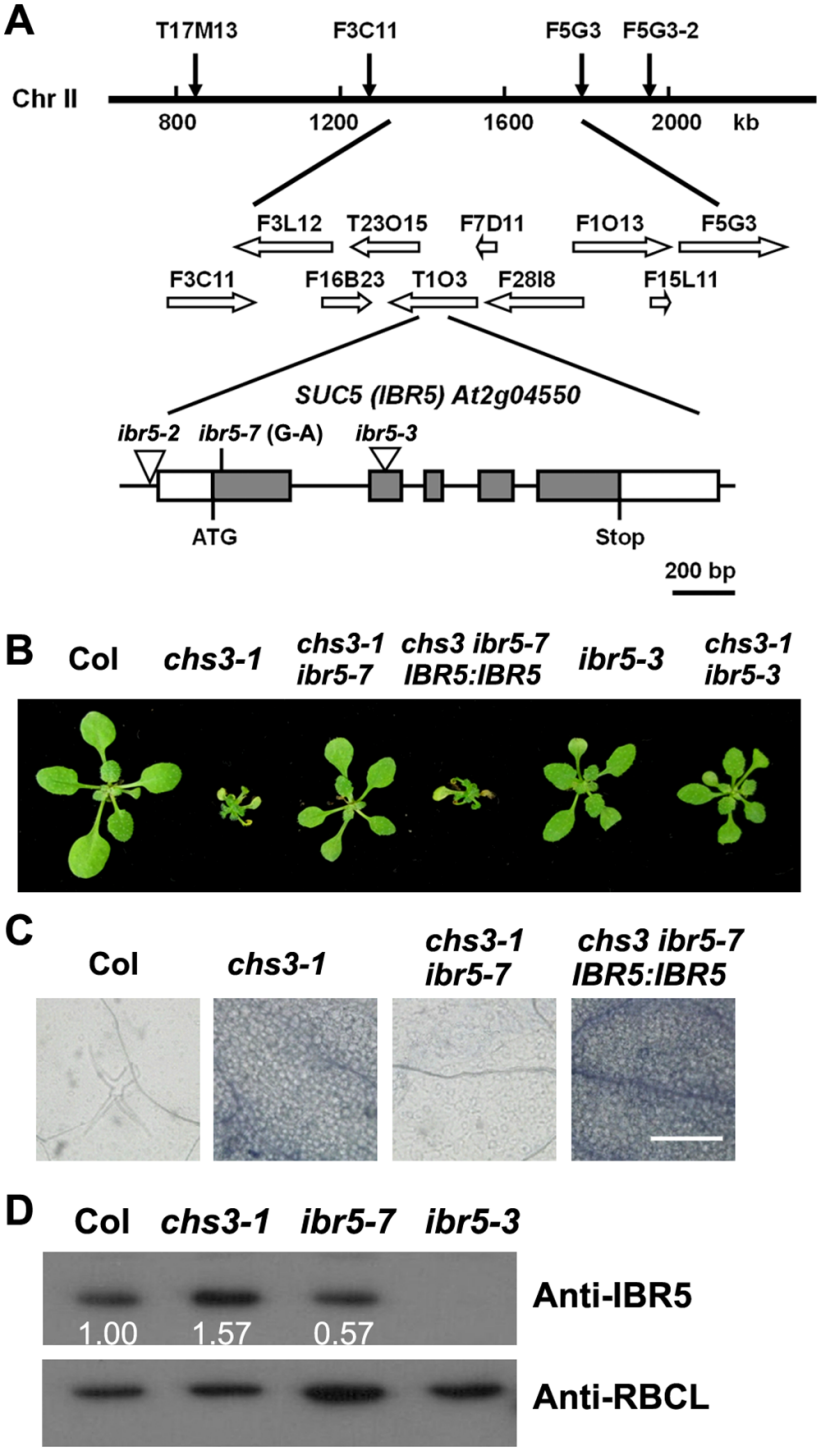
Identification of *SUC5*. (A) Map position and genomic structure of the *SUC5* gene. Exons, introns and UTRs are presented as gray boxes, lines and white boxes, respectively. The point mutation and T-DNA insertions are shown. (B) Morphology of the wild-type, *chs3-1*, *chs3-1 ibr5-7*, complemented transgenic plants (*IBR5*:*IBR5 chs3 ibr5-7*), T-DNA insertion mutant (*ibr5-3*) and *chs3-1 ibr5-3* double mutant. The photographs show 3-week-old plants grown in soil at 16°C. (C) Trypan blue staining of true leaves from wild-type, *chs3-1*, *chs3-1 ibr5-7* and *IBR5*:*IBR5 chs3-1 ibr5-7* plants. Bars: 200 μm. (D) IBR5 protein levels in wild-type, *chs3-1* and *ibr5* mutants. Total protein was extracted from 2-week-old seedlings. IBR5 protein was analyzed by immunoblotting using an anti-IBR5 antibody. Anti-RBCL was used as a loading control.

To map the *suc5* mutation, *chs3 suc5* in Columbia (Col) was crossed with Landsberg *erecta* (L*er*) to generate a mapping population. Among the F2 progeny, 50 plants homozygous or heterozygous at the *chs3-1* locus and exhibiting a wild-type morphology at 16°C were used for rough mapping. The *suc5* mutation was initially mapped to the top of chromosome II (Fig 2A). Fine mapping using approximately 200 plants refined the mutation to a 500-kb region between markers F3C11 and F5G3 (Fig 2A). Further sequencing analysis of this region in *chs3 suc5* revealed a G-to-A substitution in the first exon of *At2g04550*, resulting in an amino substitution from Arg to Lys (Fig 2A).

*At2g04550* was previously identified as *IBA RESPONSE5* (*IBR5*), which encodes a putative dual-specificity protein phosphatase [28]. To determine whether the mutation in *IBR5* is responsible for the suppression the phenotypes of *chs3*, a *chs3 ibr5-3* double mutant was generated by crossing *chs3-1* with *ibr5-3* [29]. The *ibr5-3* mutant largely restored the growth defects of *chs3-1* mutant plants grown at 16°C (Fig 2B). Furthermore, a wild-type genomic *IBR5* fragment containing 5.0 kb of the 5’-promoter region and the 3’ untranslated region was transformed into the *chs3 suc5* mutant. All eight T1 transgenic plants displayed *chs3*-conferred morphological and cell death phenotypes (Fig 2B and 2C). Taken together, these results indicate that *SUC5* is indeed *IBR5*. Therefore, *suc5* was renamed *ibr5-7*.

The *ibr5-7* single mutant was isolated by crossing *chs3 ibr5-7* with wild-type Col plants. Immunoblot analysis showed that the IBR5 protein level in *ibr5-7* was lower than that in the wild-type Col plants, whereas no IBR5 protein was detected in *ibr5-3* (Fig 2D). Intriguingly, IBR5 protein accumulated in the *chs3-1* mutant (Fig 2D), implying that IBR5 might stabilize CHS3 protein. Consistent with a previous study on *ibr5* loss-of-function mutants [28], *ibr5-7* displayed serrated leaves and was resistant to high concentrations of exogenous auxins, including IAA, IBA and 2, 4-D (S1 Fig). These results indicate that *ibr5-7* is a novel null allele of *IBR5*.

### IBR5 interacts with CHS3

**Fig 3:**
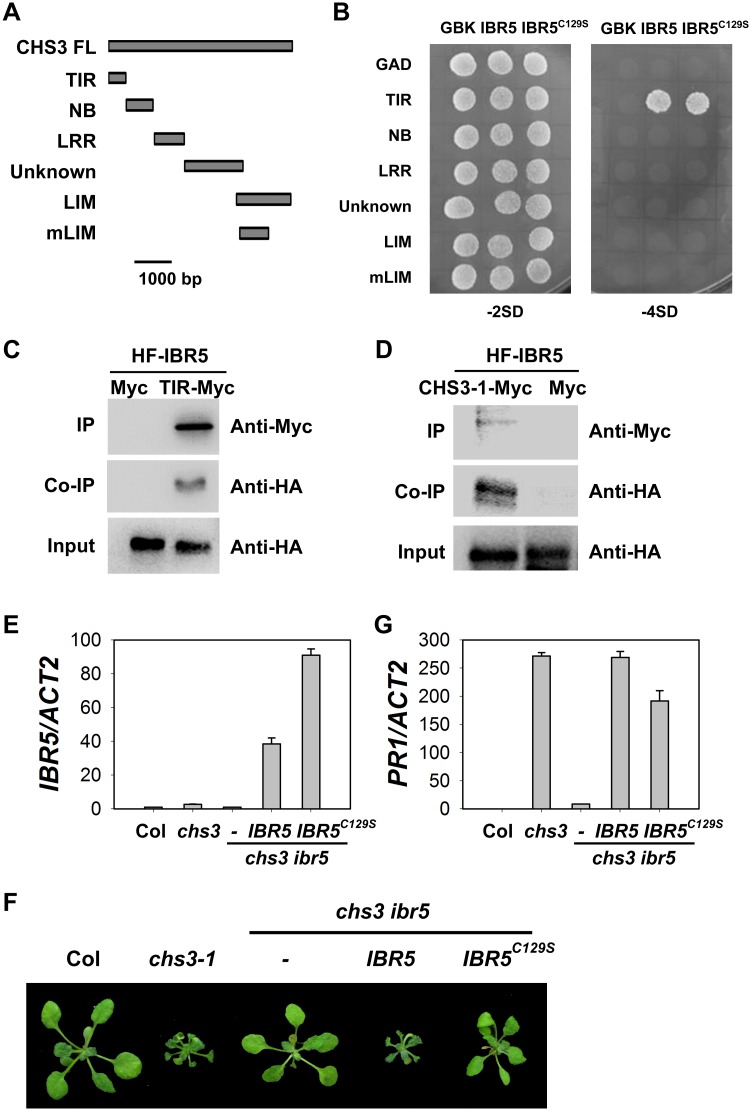
Interaction of IBR5 with CHS3. (A) Predicted protein structure of CHS3. TIR, Toll/Interleukin–1 receptor domain (aa 1–138); NB, nucleotide binding domain (aa 139–468); LRR, leucine-rich repeat domain (aa 469–729); Unknown, domain with no known function (aa 730–1240); LIM, LIM (Lin-11, Isl-1 and Mec-3 domains)-containing domain (aa 1135–1614); mLIM, LIM-containing domain with the same mutation in *chs3-1*, which contains a stop codon at 1422. (B) Interaction between IBR5 and CHS3 in yeast. The different CHS3 domains shown in (A) were fused with the pGADT7 vector. IBR5 and mutated IBR5^C129S^ were fused with the pGBKT7 vector. The constructs were transformed into AH109 yeast cells and spotted onto SD media lacking Trp and Leu (-2SD) or lacking Trp, Leu, His and Ade (-4SD). The experiments were performed four times with similar results. (C) Co-immunoprecipitation of IBR5 and the TIR domain of CHS3. Total proteins were extracted from protoplasts expressing *35S*:*HA-Flag-IBR5* (*35S*:*HF-IBR5*) with *Super*: *TIR domain of CHS3* (*Super*:*TIR-Myc*) or *Super*:*Myc*, and immunoprecipated with anti-Myc antibody. The proteins were obtained from crude lysates (Input) and the immunoprecipated proteins were detected using an anti-HA antibody. (D) Interaction of IBR5 and full-length CHS3 *in vivo*. Total proteins were extracted from *N*. *benthamiana* leaves transfected with *35S*:*HF-IBR5* and *Super*:*CHS3-1-Myc*, and were immunoprecipated with anti-Myc antibody. The proteins from crude lysates (Input) and the immunoprecipated proteins were detected using an anti-HA antibody. (E) Expression of *IBR5* in 3-week-old *Super*:*IBR5-Myc* and *Super*:*IBR5* ^*C129S*^ *-Myc* transgenic plants in *chs3 ibr5* grown at 16°C. (F) The phenotypes of *Super*:*IBR5-Myc* and *Super*:*IBR5* ^*C129S*^ *-Myc* transgenic plants in *chs3 ibr5*. The photographs show 3-week-old plants grown in soil at 16°C. (G) The expression of *PR1* in 3-week-old *Super*:*IBR5-Myc* and *Super*:*IBR5* ^*C129S*^ *-Myc* transgenic *chs3 ibr5* plants grown at 16°C.

To further investigate whether IBR5 physically interacts with CHS3 in *CHS3*-mediated signaling, a yeast two-hybrid assay was performed. As a TIR-NB-LRR-LIM-containing protein, the full-length CHS3 protein is approximately 185 kD and is difficult to express in yeast. Therefore, truncated constructs individually carrying the TIR, NB, LRR, unknown and LIM domains of CHS3 were generated and transformed into yeast (Fig 3A). IBR5 directly interacted with the TIR domain of CHS3 in yeast (Fig 3B).

Previous studies have shown that CHS3 localizes to the nucleus [24], consistent with the results of the present study (S2B Fig). We also observed that IBR5 localized to both the cytosol and the nucleus (S2A and S2B Fig). To determine whether CHS3 interacts with IBR5 *in vivo*, *35S*:*HA-Flag-IBR5* (*HF-IBR5*) and the Myc-tagged TIR domain of CHS3 driven by the Super promoter (*TIR-Myc*) were transiently expressed in *Arabidopsis* mesophyll protoplasts, and subsequently, a co-immunoprecipitation (co-IP) assay was performed. IBR5 precipitated the TIR domain of CHS3 (Fig 3C). Moreover, HF-IBR5 interacted with full-length CHS3-1-Myc (the mutated form of CHS3 containing the same mutation as the *chs3-1* mutant) when expressed in *N*. *benthamiana* (Fig 3D). These results indicated that CHS3 interacts with IBR5 through the TIR domain of CHS3 *in vivo*.

IBR5 belongs to a family of dual specificity protein phosphatases (DSPs), and the conserved cysteine in the conserved motif (VxVHCx2GxSRSx5AYLM) of DSPs is necessary for catalytic activity in many DSP proteins, including IBR5 and its homolog DsPTP1 [30]. To examine whether the phosphatase activity of IBR5 is required for interactions with CHS3, we generated the mutated form of IBR5 (IBR5^C129S^). The yeast two-hybrid assay showed that the mutation did not affect the interaction of IBR5 and CHS3 (Fig 3B), suggesting that the catalytic activity is not necessary for the interaction between IBR5 and CHS3. We next introduced *Super*:*IBR5-Myc* and *Super*:*IBR5* ^*C129S*^ *-Myc* into the *chs3 ibr5* mutant background (Fig 3E, 3F and 3G). As a control, *IBR5-Myc* fully recovered the *chs3 ibr5* phenotype and *PR1* expression. However, the morphological phenotypes and *PR1* expression of *chs3 ibr5* were partially rescued in the mutated *IBR5* ^*C129S*^ *-Myc* plants (Fig 3F and 3G). These results suggest that the catalytic activity of IBR5 is required for full function in the CHS3-mediated defense response.

### IBR5 promotes CHS3 accumulation at both transcription and protein levels

**Fig 4:**
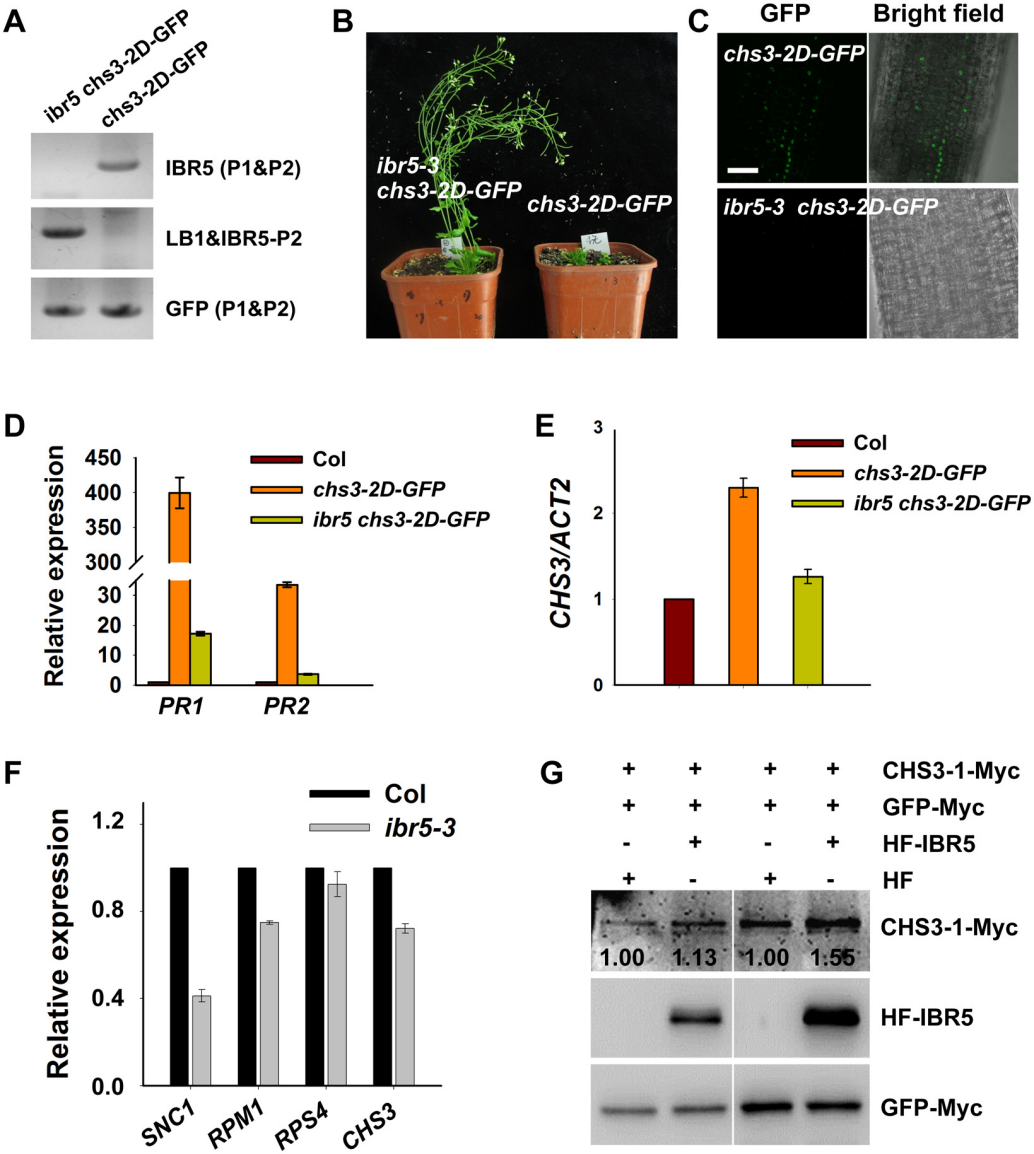
CHS3 is promoted by *IBR5* at both mRNA and protein levels. Genotyping of *ibr5-3 chs3-2D-GFP* plants. The primers used for genotyping the *chs3-2D-GFP* gene were GFP-P1 and GFP-P2, and the primers used for genotyping *ibr5-3* were IBR5-P1, IBR5-P2 and LB1 as listed in S1 Table. The phenotype of *chs3-2D-GFP* transgenic plants was rescued by *ibr5-3*. *chs3-2D-GFP* and *ibr5-3 chs3-2D-GFP* plants were grown for 5 weeks, and representative plants are shown. Expression of CHS3-2D-GFP protein in 8-d-old *chs3-2D-GFP* and *ibr5-3 chs3-2D-GFP* plants. Bar: 100 μm. (D) Expression of CHS3-2D-GFP protein in 8-day-old *chs3-2D-GFP* and *ibr5-3 chs3-2D-GFP* plants. Bar: 100 μm. (D, E) Expression of the *PR1*, *PR2* (D) and *CHS3* (E) genes in *chs3-2D-GFP* and *ibr5-3 chs3-2D-GFP* plants. The values were normalized to the expression of *ACTIN2*. The error bars represent the SD of three replicates. (F) The expression of several *R* genes (*SNC1*, *RPM1*, *RPS4* and *CHS3*) in 10-day-old *ibr5-3* mutant grown at 22°C. The values were normalized to the expression of *ACTIN2*. The error bars represent the SD of three replicates. Similar results were obtained in three independent experiments. (G) The effect of IBR5 on the CHS3 protein level in *Arabidopsis* protoplasts. *Super*:*CHS3-1-Myc* and *35S*:*HF-IBR5* or *35S*:*HF* constructs were co-expressed in *Arabidopsis* protoplasts. IBR5 and CHS3 proteins were detected using anti-HA and anti-Myc antibodies. The *GFP-Myc* construct was used as a control for transformation efficiency. The numbers show the ratios of CHS3-1-Myc to GFP-Myc protein levels. Two biological repeats are shown with similar results.

Another gain-of-function *chs3* mutant, *chs3-2D*, was dwarfed and exhibited a constitutively active defense phenotype at 22°C [24]. Moreover, *chs3*:*chs3-2D-GFP* transgenic plants were generated, exhibiting *chs3-2D-*like phenotypes [24]. To examine whether *IBR5* affects the accumulation of CHS3 in *chs3-2D-GFP* plants, *ibr5-3 chs3-2D-GFP* plants were generated by crossing *ibr5-3* with *chs3-2D-GFP* plants (Fig 4A). The dwarf phenotype of *chs3-2D-GFP* plants was fully restored by *ibr5-3* (Fig 4B), and GFP signals were observed in the nuclei of *chs3-2D-GFP* transgenic plants (Fig 4C). However, no obvious GFP signal was detectable in *ibr5-3 chs3-2D-GFP* plants (Fig 4C). Consistently, the expression of *PR* genes in *chs3-2D-GFP* was also suppressed in *ibr5-3* plants (Fig 4D). The expression of *CHS3* in *ibr5-3 chs3-2D-GFP* plants was also examined. As shown in Fig 4E, *CHS3* expression in *ibr5-3 chs3-2D-GFP* plants was dramatically decreased compared with *chs3-2D-GFP* plants, consistent with the result obtained in *chs3 ibr5-7* (Fig 1F). Moreover, the expression of *CHS3* was down-regulated in the *ibr5-3* mutant (Fig 4F). These results suggest that IBR5 positively regulates the expression of *CHS3*, which might at least be partially responsible for the decreased CHS3-2D-GFP signals in *ibr5-3 chs3-2D-GFP*.

To dissect the influence of IBR5 on CHS3 at the protein level, we examined the effect of IBR5 on the CHS3 protein level in *Arabidopsis* protoplasts. The protein level of CHS3-1-Myc in protoplasts co-expressing *CHS3-1-Myc* and *HF-IBR5* was much higher than that in protoplasts expressing *CHS3-1-Myc* and *HF* (Fig 4G). This result suggests that IBR5 also promotes the accumulation of CHS3 protein in plant.

### *HSP90* and *IBR5* synergistically regulate *chs3*-conferred phenotypes

**Fig 5:**
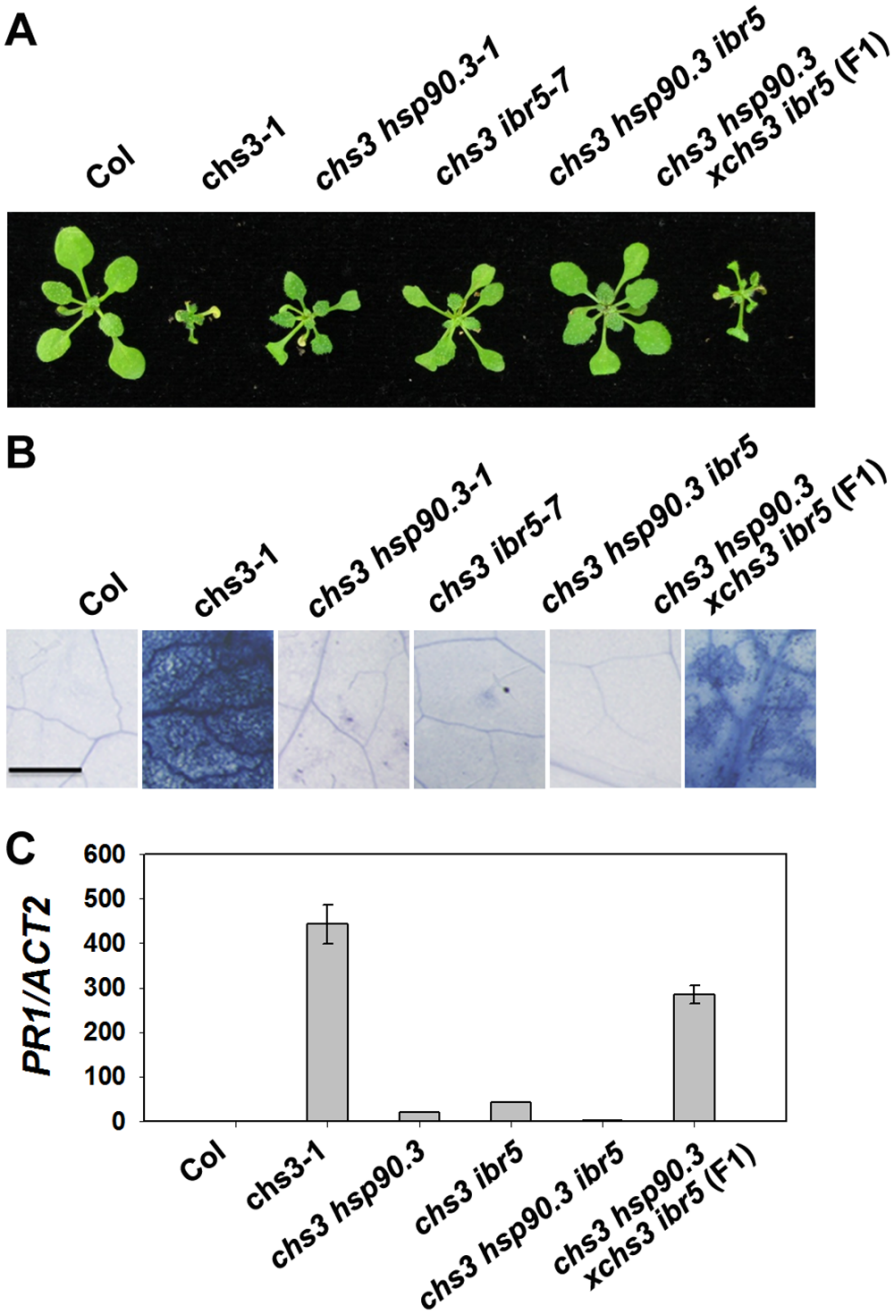
Genetic analysis of *CHS3*, *IBR5* and *HSP90*.*3*. (A) Morphology of 4-week-old wild-type Col, *chs3-1*, *chs3 hsp90*.*3–1*, *chs3 ibr5-7 chs3 hsp90*.*3 ibr5* and F1 progeny of *chs3 hsp90*.*3–1* crossed with *chs3 ibr5-7* grown in soil at 16°C. (B) Trypan blue staining of the leaves from the plants described in (A). Bar: 50 μm. (C) *PR1* gene expression in the plants described in (A). The values were normalized to the expression of *ACTIN2*. The error bars represent the SD of three replicates. The experiments were performed three times with similar results.

HSP90 plays an important role in the stability of R proteins [31]. A previous study showed that the *hsp90*.*3–1* mutant was a suppressor of the *chs2/rpp4-1d* mutant [32]. Therefore, we examined whether *hsp90*.*3–1* rescues the chilling-sensitive phenotype of *chs3-1*. The *chs3-1 hsp90*.*3–1* double mutant was generated and largely showed wild-type morphology but was smaller at 16°C (Fig 5A), and cell death was dramatically suppressed (Fig 5B). *PR1* expression was also significantly inhibited in the double mutant (Fig 5C). Further analysis showed that the F1 progeny of *ibr5 chs3* and *hsp90*.*3 chs3* could partially rescue the morphology, cell death and *PR1* gene expression of *chs3* (Fig 5A, 5B and 5C). Moreover, the *chs3 hsp90*.*3 ibr5* triple mutant more closely resembled wild-type plants than the *chs3 hsp90* and *chs3 ibr5* mutants in terms of growth, cell death and *PR1* gene expression (Fig 5A, 5B and 5C). These data indicate that HSP90 and IBR5 synergistically modulate the *chs3*-conferred growth and defense responses.

### IBR5 interacts with HSP90 and SGT1b

**Fig 6:**
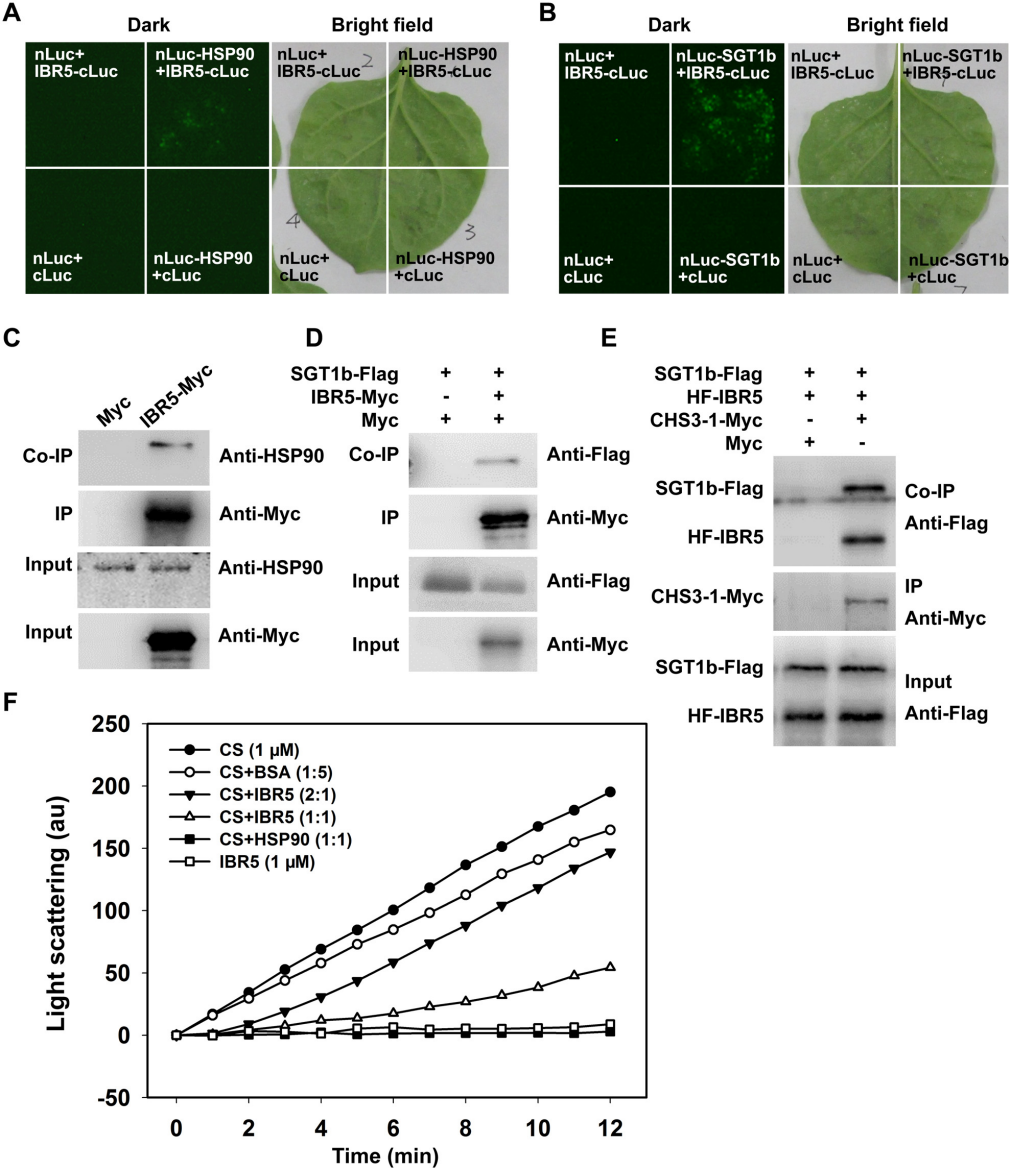
Interaction of IBR5 with HSP90 and SGT1b. (A) Interaction of IBR5 with HSP90 as revealed via firefly luciferase complementation imaging assay in *N*. *benthamiana* leaves. nLuc+cLuc-IBR5, HSP90-nLuc+cLuc, and nLuc+cLuc were used as negative controls. (B) Interaction of IBR5 with SGT1b as revealed by a firefly luciferase complementation imaging assay in *N*. *benthamiana* leaves. nLuc+cLuc-IBR5, SGT1b-nLuc+cLuc, and nLuc+cLuc were used as negative controls. (C) Co-immunoprecipitation of IBR5 and HSP90. Total protein was extracted from 2-week-old transgenic seedlings expressing *Super*:*IBR5-Myc* or *Super*:*Myc*. Anti-Myc antibody was used for immunoprecipitation, and the immunoprecipitated proteins were analyzed by immunoblotting using an anti-HSP90 antibody. (D) Co-immunoprecipitation of IBR5 and SGT1b. Total protein was extracted from transgenic plants expressing *Super*:*Myc/Super*:*SGT1b-Flag* or *Super*:*IBR5-Myc/Super*:*SGT1b-Flag*. An anti-Myc antibody was used for immunoprecipitation, and the immunoprecipitated proteins were analyzed by immunoblotting using an anti-Flag antibody. (E) Interaction of CHS3 with IBR5 and SGT1b in plant. Total proteins were extracted from *N*. *benthamiana* leaves transfected with *35S*:*HF-IBR5*, *Super*:*SGT1b-Flag* and *Super*:*CHS3-1-Myc*, and were immunoprecipated with anti-Myc antibody. The proteins from crude lysates (Input) and the immunoprecipated proteins were detected using an anti-Flag antibody. (F) IBR5 can stabilize CS under thermally denaturing conditions. Citrate synthase (1 μM) was incubated alone or with 5 μM BSA (1:5), 0.5 μM IBR5 (2:1), 1 μM IBR5 (1:1) or 1 μM HSP90 (1:1) at 43°C. The kinetics was determined by light scattering using a fluorescence spectrophotometer.

HSP90 functions as a complex with SGT1b and RAR1 [11,33–35] and we previously showed that *sgt1b* and *rar1* suppressed the phenotypes of *chs3-1* [23], similar to *ibr5* (Fig 1). Next we determined whether IBR5 physically interacts with HSP90 and SGT1b in plants. To this end, a luciferase complementation imaging (LCI) assay was performed in *Nicotiana benthamiana*. IBR5 interacted with HSP90 and SGT1b in *N*. *benthamiana* leaves (Fig 6A and 6B). Furthermore, co-IP assays showed that endogenous HSP90 successfully immunoprecipitated IBR5-Myc in transgenic plants expressing *IBR5-Myc*, but not in *Myc* transgenic plants (Fig 6C). Similarly, SGT1b could be co-immunoprecipitated with IBR5 in transgenic plants expressing *IBR5-Myc* and *SGT1b-FLAG*, but not in transgenic plants expressing *Myc* and *SGT1b-FLAG* (Fig 6D). We also examined the association between SGT1b and CHS3. Co-IP assays showed that SGT1b associated with the TIR domain of CHS3 and full-length CHS3-1 *in vivo* (S3 Fig). Furthermore, we examined the interaction of CHS3-1 with IBR5 and SGT1b in *N*. *benthamiana* leaves. As shown in Fig 6E, CHS3-1 could simultaneously pull down IBR5 and SGT1b in plants. These results indicate that IBR5 forms complex(es) with CHS3, HSP90 and SGT1b *in vivo*.

### IBR5 exhibits holdase activity *in vitro*

We subsequently investigated whether IBR5 protects proteins *in vitro* using a thermal aggregation assay with citrate synthase (CS) as a model substrate [36]. CS aggregation was examined after measuring the absorbance at 500 nm under thermally denaturing conditions (40°C or above). Heat-induced aggregation of CS was inhibited by HSP90 (Fig 6F), consistent with the results of previous study [37]. IBR5 showed a weaker but more significant effect than HSP90 on the inhibition of CS aggregation. When IBR5 was incubated with CS at 43°C, the aggregation of CS was partially suppressed in a dose-dependent manner (Fig 6F). This result indicates that IBR5 protects proteins against aggregation *in vitro*.

### Genetic and physical interactions between IBR5 and SNC1

**Fig 7:**
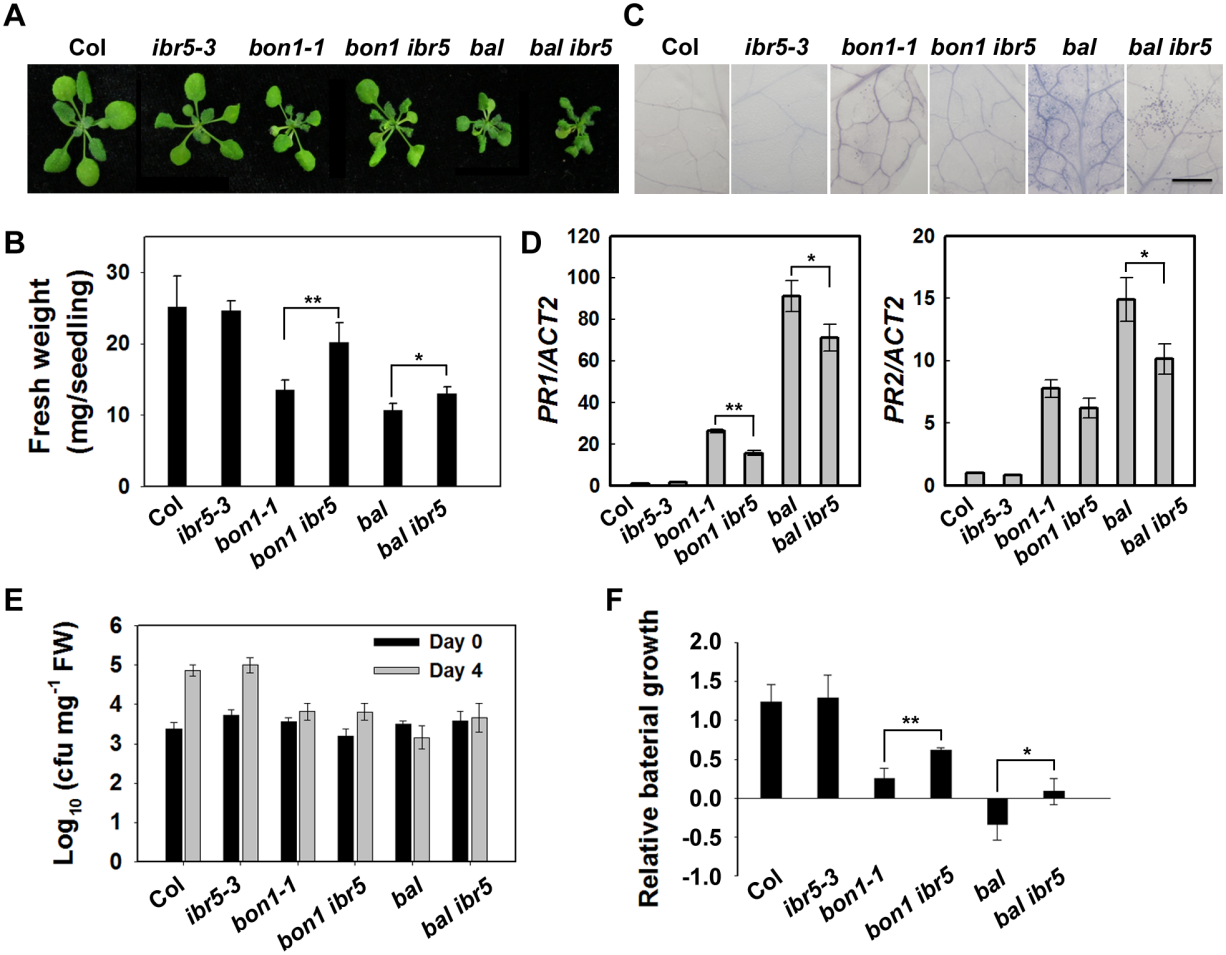
The defense phenotypes of *bon1-1 ibr5-3* and *bal ibr5-3*. Morphology of 3-week-old Col, *ibr5-3*, *bon1-1*, *bon1-1 ibr5-3*, *bal*, *bal ibr5-3* grown at 22°C. (B) Fresh weight of plants in (A). The error bars represent the SD of three replicates, and the asterisks indicate significant differences (**P < 0.01, *P < 0.05, *t*-test). (C) Trypan blue staining of leaves from the plants described in (A). Bar: 0.5 mm. The expression of *PR* genes in plants in (A). The error bars represent the SD of three replicates, and the asterisks indicate significant differences (**P < 0.01, *P < 0.05, *t*-test). (E) The effect of IBR5 on pathogen resistance of *bon1-1* and *bal* to *P*.*s*.*t*. DC3000. Three-week-old seedlings were dipped with *P*.*s*.*t*. DC3000 (OD_600_ = 0.05), and leaves were taken immediately (day 0) and at day 4 after infection. The log-transformed values presented are the mean values of three replicates ± SD. The experiments were performed three times with similar results. (F) Relative bacterial growth of plants in (E) after infection with *P*.*s*.*t*. DC3000. The values are the ratios of bacteria number at day 4 to day 0. The error bars represent the SD of three replicates, and the asterisks indicate significant differences (**P < 0.01, *P < 0.05, *t*-test).

The TIR domain of CHS3 shares amino acid similarity with TIR-NB-LRR R proteins SNC1 and RPP4 (S4 Fig). Gain-of-function mutants of *SNC1* (*snc1-1* and *bal/snc1-2*) activate the defense response in a temperature-dependent manner [6,38]. The loss of *BON1* function in *Arabidopsis* leads to temperature-sensitive growth and autoimmune phenotypes resulting from the activation of SNC1 [6]. Phenotype analyses showed that the *bon1-1 ibr5-3* (*bon1 ibr5*) and *bal ibr5-3* (*bal ibr5*) double mutants were slightly larger than the *bon1-1* and *bal* single mutants (Fig 7A and 7B). Moreover, the cell death in *bon1 ibr5* and *bal ibr5* was reduced (Fig 7C). Consistently, the expression of *PR1* and *PR2* genes in *bon1 ibr5* and *bal ibr5* was also lower than that in the *bon1-1* and *bal/snc1-2* mutants (Fig 7D). We also examined the basal defense response of *bon1 ibr5* and *bal ibr5* to *P*.*s*.*t*. DC3000. The pathogen resistance of *bon1-1* and *bal/snc1-2* mutants to *P*.*s*.*t*. DC3000 was slightly suppressed in the *ibr5-3* mutant (Fig 7E and 7F). These genetic data suggest that IBR5 is partially implicated in the SNC1-mediated defense response.

**Fig 8:**
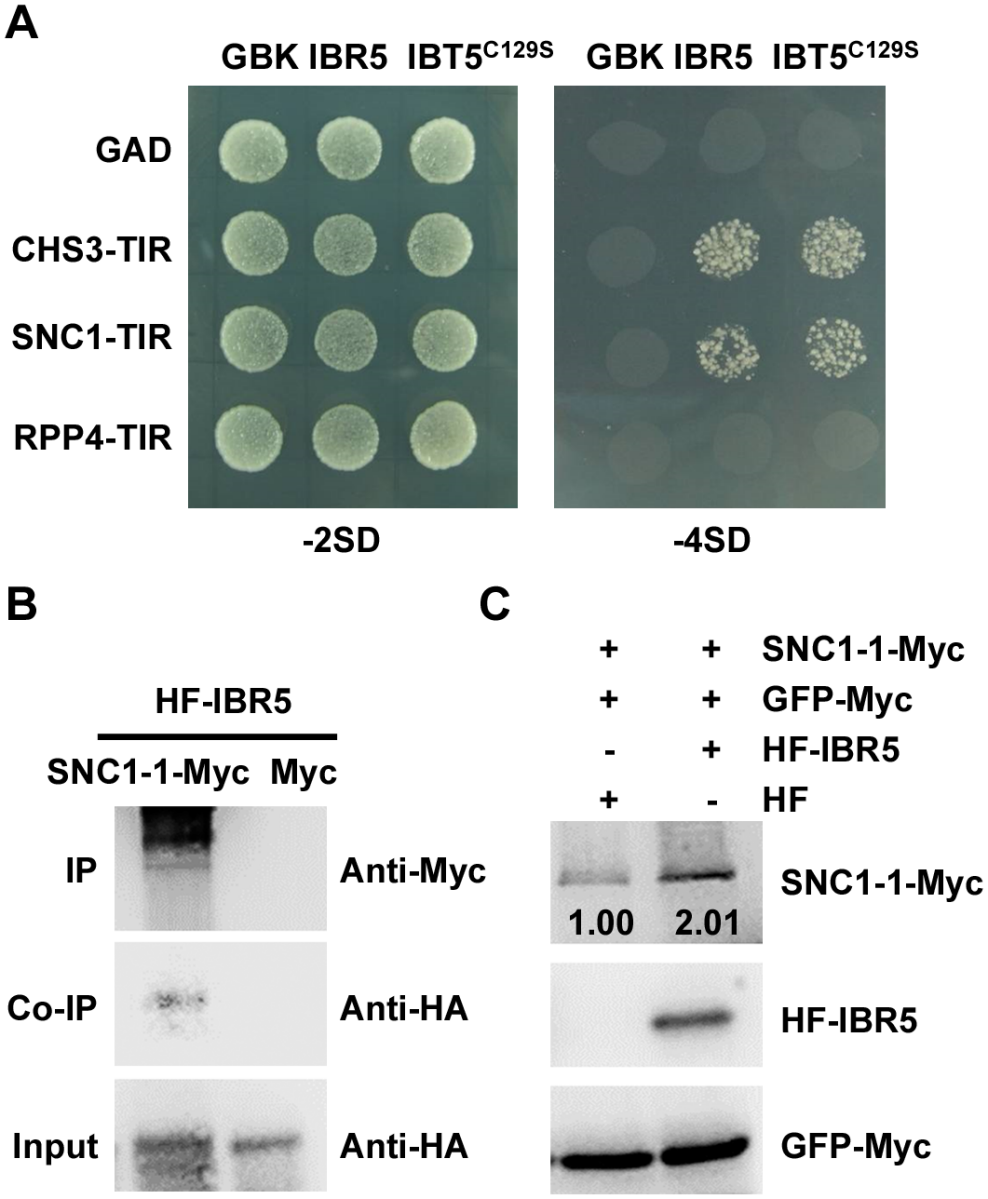
Interaction of IBR5 with SNC1 and RPP4. (A) Interaction of IBR5 with TIR domains of CHS3, SNC1 and RPP4 in yeast. The experiments were performed three times with similar results. (B) Interaction of IBR5 and full-length SNC1 *in vivo*. Total proteins were extracted from *N*. *benthamiana* leaves transfected with *35S*: *HF-IBR5* and *Super*:*SNC1-1-Myc* and were immunoprecipated with an anti-Myc antibody. The proteins from crude lysates (Input) and the immunoprecipated proteins were detected using an anti-HA antibody. (C) The effect of IBR5 on SNC1 protein level in *Arabidopsis* protoplasts. *Super*:*SNC1-1-Myc* and *35S*:*HF-IBR5* or *35S*:*HF* constructs were co-expressed in *Arabidopsis* protoplasts. IBR5 was detected with anti-HA antibody and SNC1-1 was detected using an anti-Myc antibody. *GFP-Myc* construct was used as a control for transformation efficiency.

To further dissect the function of IBR5 on SNC1, we examined the expression of *SNC1* in the *ibr5-3* mutant. As shown in Fig 4F, the *SNC1* transcript levels in the *ibr5-3* mutant were much lower than those in Col, indicating that *SNC1* expression is positively regulated by IBR5. We next explored whether IBR5 physically interacts with SNC1. A yeast two-hybrid assay showed that IBR5 interacted with the TIR domain of SNC1 (Fig 8A). The interaction of IBR5 and full-length SNC1-1 (the mutated form of SNC1 containing the same mutation as the *snc1-1* mutant) was confirmed by a co-IP assay in *N*. *benthamiana* leaves (Fig 8B). Moreover, the protein level of SNC1-1-Myc increased when co-expressed with HF-IBR5 in *Arabidopsis* protoplasts (Fig 8C). Taken together, these data suggest that IBR5 might also promote SNC1 protein accumulation.

Our previous studies showed that the mutations in R protein CHS2/RPP4 or R-like protein CHS1 induce plant sensitivity and activate defense responses under chilling stress [7,8]. To investigate whether *ibr5* also suppresses the chilling sensitivity of *chs1-2* and *chs2-1/rpp4-1d*, we generated *chs1-2 ibr5-3* (*chs1 ibr5*) and *chs2-1 ibr5-4* (*chs2 ibr5*) double mutants. In terms of morphology and cell death, *chs1 ibr5* and *chs2 ibr5* mutants showed *chs1-2* and *chs2-1* single mutant phenotypes under chilling stress (S5A and S5B Fig). However, *PR1* expression in *chs1 ibr5* was partially suppressed, whereas the expression of this protein in *chs2 ibr5* was not obviously affected. A yeast two-hybrid assay showed that IBR5 did not interact with RPP4 (Fig 8A). We also examined the pathogen resistance of *ibr5* mutants to RPP4-specific *Hyaloperonospora arabidopsidis* (*H*.*a*.) Emwa1. As controls, Col was completely resistant to *H*.*a*. *Emwa1*, whereas *rpp4-r26*, which contains a mutation in *RPP4* resulting in the introduction of a stop codon in front of the LRR domain [32], was susceptible to *H*.*a*. *Emwa1* (S6 Fig). The *ibr5-3* and *ibr5-7* mutants showed complete resistance to *H*.*a*. *Emwa1* (S6 Fig), suggesting that IBR5 might not be involved in RPP4-mediated oomycete resistance.

### IBR5 is involved in RPM1- and RPS4-mediated disease resistance

**Fig 9:**
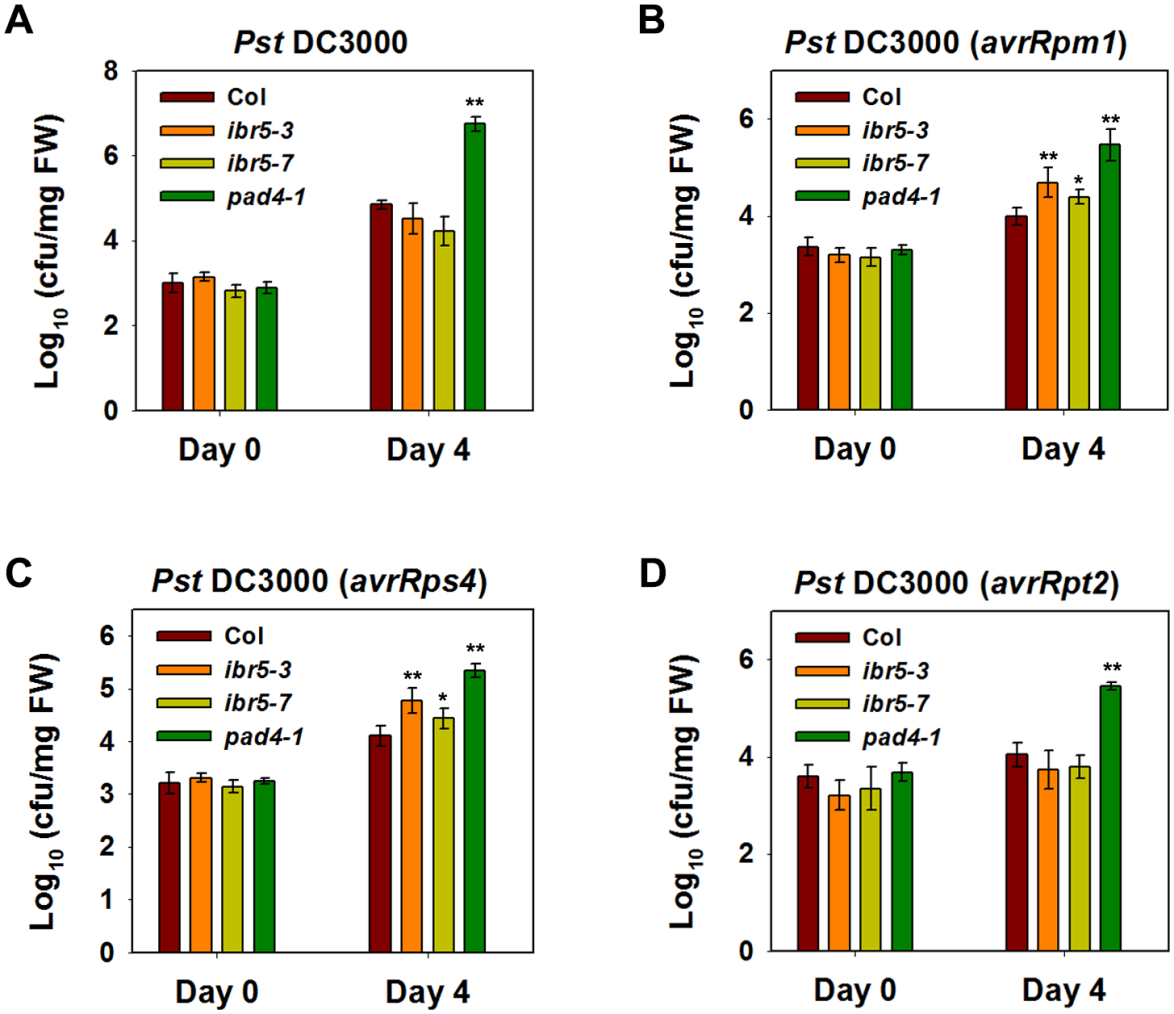
Pathogen resistance analysis of *ibr5* mutants. (A) Response of *ibr5* to *P*.*s*.*t*. DC3000. Three-week-old plants were dipped with *P*.*s*.*t*. DC 3000 (OD_600_ = 0.05), and leaves were taken immediately (day 0) and at day 4 after infection. The log-transformed values presented are the mean values of three replicates ± SD. (B-D) Responses of *ibr5* to avirulent bacteria. Three-week-old plants were dipped with *P*.*s*.*t*. DC3000 (*avrRpt2*) (B), *P*.*s*.*t*. DC3000 (*avrRpm1*) (C) and *P*.*s*.*t*. DC3000 (*avrRps4*) (D) (OD_600_ = 0.2). In (A-D), the log-transformed values presented are the mean values of three replicates ± SD, and the asterisks indicate significant differences compared with wild-type Col (**P < 0.01, *P < 0.05, *t*-test). All experiments were repeated at least three times with similar results.

To examine whether basal defense was affected in the *ibr5* single mutant, we infected wild-type Col and *ibr5* mutants with virulent *P*.*s*.*t*. DC3000. The bacterial growth in *ibr5-3* and *ibr5-7* was comparable to that in wild-type plants, whereas as the control, *pad4-1*, supported 100 times more bacterial growth than the wild-type plants (Fig 9A), suggesting that IBR5 does not play an important role in basal defense.

As the role of CHS3 is compromised in the *ibr5* mutant, we further investigated whether IBR5 is required for other *R*-gene-mediated disease resistance. The *ibr5* mutants were inoculated with *P*.*s*.*t*. DC3000 carrying either *avrRpm1*, *avrRps4*, or *avrRpt2*. The *ibr5* mutants showed enhanced susceptibility to *P*.*s*.*t*. DC3000 (*avrRpm1*) and *P*.*s*.*t*. DC3000 (*avrRps4*) (Fig 9B and 9C). In contrast, no remarkable difference in the growth of *P*.*s*.*t*. DC3000 (*avrRpt2*) was detected between the *ibr5* mutants and wild-type Col (Fig 8D). As a control, *pad4-1* showed enhanced disease susceptibility to all three avirulent bacterial strains (Fig 9B, 9C and 9D). These results suggest that IBR5 might also contribute to disease resistance mediated by RPM1 and RPS4.

We next examined the expression of *RPM1* and *RPS4* in the *ibr5-3* mutant. The transcription levels of *RPM1* and *RPS4* were slightly down-regulated in the *ibr5-3* mutant compared with wild type plants (Fig 4F). Furthermore, we examined whether IBR5 interacts with TIR-NB-LRR protein RPS4 or CC-NB-LRR protein RPM1. The results of a yeast two-hybrid assay showed that neither wild-type IBR5 nor mutated IBR5^C129S^ interacted with the TIR domain of RPS4 or CC domain of RPM1 (S7A Fig). However, interestingly, we observed the direct interaction of IBR5 and IBR5^C129S^ with RPS4-interacting protein RRS1 [39] in yeast (S7B Fig). The results of additional co-IP assays showed that IBR5 could pull down RPS4 (S7C Fig), suggesting that IBR5 might form a complex with RPS4 and RRS1. In contrast, no interaction between IBR5 and RPM1 was observed in plants (S7D Fig). Furthermore, no obvious change in the RPM1 protein level was detected when IBR5 and RPM1 were co-expressed (S7E Fig).

## Discussion

CHS3 is involved in temperature-dependent defense responses [23]. In the present study, we identified *ibr5-7* as a suppressor of *chs3-1*. IBR5 interacts with CHS3 and HSP90/SGT1b chaperones to stabilize the CHS3 protein, thereby modulating temperature-dependent defense responses. In addition, IBR5 is invovled in defense responses mediated by several other *R* genes, including *SNC1*, *RPS4* and *RPM1*.

IBR5 is a MAP kinase phosphatase (MKP) that dephosphorylates activated MAPKs [40,41]. Emerging evidence has shown that MKPs play important roles in modulating plant defense responses [41]. For example, the *mkp1* null mutation in the Col accession exhibits constitutive stress response phenotypes, causing a dwarf stature dependent on SNC1 [42]. However, it remains unclear whether the substrates of MKP1, MPK3 and MPK6 [42,43] are involved in the regulation of SNC1. Additional studies have revealed that MKP1 functions as a negative regulator of MPK6-mediated pathogen-associated molecular pattern (PAMP) responses and disease resistance [44]. The *mkp2* null mutant displays delayed symptoms of disease induced by *Ralstonia solanacearum*, and shows increased susceptibility to *Botrytis cinerea* [45]. MKP2 might regulate MPK3/MPK6 activity during different pathogen defense responses. Moreover, MKP2 reverses hypersensitive-like responses triggered by MPK6 upon plant treatment with fungal elicitors [45,46]. It is likely that these MKPs have negative functions in defense signaling pathways through their MPK substrates. The data obtained in the present study showed that the loss of *IBR5* function suppresses *chs3*-conferred, temperature-dependent defense responses and slightly weakens the autoimmunity of *bal/snc1-2*, a gain-of-function mutant of a TIR-NB-LRR protein SNC1. Moreover, resistance mediated by R proteins, such as RPS4 and RPM1, is compromised in the *ibr5* mutants. These results suggest that IBR5 plays a positive role in the ETI pathway mediated by multiple R proteins. Therefore, different MKPs play either positive or negative roles in plant immunity. As IBR5 is a MAP kinase phosphatase, we also examined whether the dephosphorylation activity of IBR5 is required for the regulation of CHS3. The inactive form of IBR5 (IBR5^C129S^) partially rescues the phenotype of *chs3 ibr5*, suggesting that the dephosphorylation activity of IBR5 is necessary for the complete function of this protein in the CHS3-mediated defense response.

MPK12 is a substrate of IBR5 [29], and the reduced expression of *MPK12* partially complements auxin, but not ABA insensitivity, in the *ibr5* mutant, suggesting that there might be other substrate(s) of IBR5 involved in IBR5-mediated ABA responses [29]. In the present study, the loss-of-function mutant *mpk12-3* displayed an auxin-sensitive phenotype (S8A, S8B and S8C Fig), similar to that of *mpk12 RNAi* lines [29]. However, *mpk12-3* cannot rescue the phenotype of *chs3 ibr5* (S8D Fig), indicating that the CHS3-mediated defense response does not require MPK12. Moreover, the mutant of auxin receptor *tir1* enhances the auxin insensitive phenotype of *ibr5*, implying that IBR5 is involved in the auxin response independently of the TIR1-mediated auxin signaling pathway [30]. Therefore, the role of IBR5 in R-mediated resistance might not occur through the function of IBR5 in the auxin response. *ibr5* mutant is shown to be insensitive to ABA, but the underlying mechanism is completely unknown [28]. ABA signaling plays a role in defense responses in a complicated manner. Recent studies have reported that the overexpression of ABA receptors maintains stomatal closure during *P*.*s*.*t*. DC3000 infections, leading to enhanced resistance after dipping, but enhanced susceptibility to the pathogen after infiltration [47]. In contrast, several ABA signaling mutants, such as *abi1* and *abi4*, do not exhibit prominent phenotypes under the *snc1-1* background or during pathogen infection [48]. The ABA-insensitive mutant *cpr22*, containing a deletion on two cyclic nucleotide-gated ion channel genes, shows constitutive *PR* gene expression, enhanced pathogen resistance and SA accumulation [49]. WRKY40, WRKY18, and WRKY60 play negative roles in ABA signaling [50]. The single and triple mutants show diverse phenotypes to different pathogens, as these mutants are more resistant to *P*. *syringae* but more susceptible to *B*. *cinerea* compared with the wild type [50]. Whether the role of IBR5 in the ABA response is involved in CHS3-mediated defense pathway remains unknown.

In plants, the TIR domain is indispensable for defense signal transduction [51]. For example, the transient expression of the TIR domain of RPS4 in *N*. *benthamiana* leaves triggers cell death in a manner dependent on both SGT1 and HSP90 [13,51]. The TIR domain of L6, a TIR-NB-LRR R protein in flax, forms a homodimer required for immune signaling [52]. A recent study showed that TIR domain heterodimerization is necessary to form a functional RRS1/RPS4 effector recognition complex [9], indicating the importance of the TIR domain of R proteins in signal recognition and transduction. Moreover, the TIR domain of R proteins interacts with other proteins [39,53–56]. In the present study, we observed that the TIR domain of CHS3 interacts with IBR5 *in vitro* and *in vivo*. The mutation of IBR5^C129S^ does not affect this interaction, suggesting that the dephosphatase activity of IBR5 is not a prerequisite for the interaction between IBR5 and CHS3. We also examined the interaction between IBR5 and four other TIR-NB-LRR R proteins: SNC1, RPP4, RPS4 and its interacting R protein RRS1 [39], and CC-NB-LRR, RPM1. Under these conditions, IBR5 interacts with SNC1 and RRS1/RPS4 in plant, but not with RPP4 and RPM1. Consistently, *ibr5* partially suppresses the cell death and *PR* gene expression in *bal/snc1-2* but does not suppress the expression of these proteins in *chs2/rpp4-1d*. These data suggest that IBR5 might specifically interact with certain TIR-NB-LRR proteins to regulate defense signaling. However, the mechanism underlying the involvement of IBR5 in defense responses mediated by the CC-type R protein remain unclear.

Increasing evidence has shown that HSP90, SGT1 and RAR1 form a protein complex with chaperone activity required for the proper folding and stabilization of R proteins [11,23,33,34,57–60]. In this complex, HSP90 and RAR1 increase R protein levels [57]. The phosphatase PP5 interacts with HSP90 and the tomato R protein I–2, and together with HSP90, PP5 acts as a co-chaperone [61]. In the present study, we found an interaction between the phosphatase IBR5 and the HSP90/SGT1B/RAR1 complex *in vivo*, suggesting that IBR5 complexes with these chaperone proteins and stabilizes CHS3. Increasing evidence further supports the notion: (1) The *in vitro* holdase activity of IBR5 indicates that this enzyme markedly inhibits temperature-dependent CS degradation. (2) The F1 progeny of *chs3 ibr5* and *chs3 hsp90* partially inhibits the *chs3* phenotype. Moreover, the *chs3 ib5 hsp90* triple mutant is larger than both *chs3 hsp90* and *chs3 ibr5* and is reminiscent of the wild type. We speculate that different complexes comprising IBR5 and HSP90 might play roles in CHS3-mediated signaling. This involvement might reflect the dosage effect of *IBR5* and *HSP90*. A similar mechanism was observed for the COI1 (CORONATINE INSENSITIVE 1) and HSP90 proteins in regulating RPM1-mediated disease resistance [62]. (3) *chs3*-dependent phenotypes are suppressed by *sgt1b*, *rar1* [23] and *hsp90*.*3–1*, indicating that HSP90, RAR1 and SGT1b are required for *CHS3* activation. (4) IBR5 interacts with the HSP90, SGT1b and CHS3 proteins *in vivo*. Collectively, these data show that IBR5 associates with HSP90/RAR1/SGT1b to stabilize the CHS3 protein. Because SGT1b interacts with a core component of the SKP1-CULLIN1-F-box (SCF) E3 ligase complex, S-phase kinase-associated protein 1 (SKP1), SGT1b has been considered as a subunit of the SCF complex [63,64]. Recent studies have shown that SNC1 is degraded by the SCF^CPR1^ complex [65,66]. In a protein-protein interactome study, IBR5 was also shown to interact with SKP1 [67]. These results suggest that IBR5, in concert with HSP90/SGT1B proteins, might affect the function of SCF E3 ubiquitin ligase complexes, thereby regulating the ubiquitination and degradation of some R proteins. Further studies will elucidate the molecular mechanisms underlying the roles of IBR5 in R-mediated defense responses.

## Materials and Methods

### Plant materials and growth conditions

*Arabidopsis thaliana* Col–0, *chs3-1* [23], *ibr5-3* [29], *pad4-1* [68], *hsp90*.*3–1*, *rpp4-r26* [32], and *mpk12-3* (SAIL_543_F07) were used in this study. Plants were grown in soil or on Murashige and Skoog (MS) medium containing 2% (w/v) Suc and 0.8% (w/v) agar at 16°C or 22°C with a 16-h light/8-h dark cycle under white light. Salicylic acid measurement and were described previously [23].

### Suppressor screening and map-based cloning of *SUC5*

The suppressors of *chs3-1* were screened and mapped as described previously [23]. Approximately 200 plants with *chs3-1* background with wild-type morphology at 16°C were used for mapping to a 500-kb region between markers F3C11 and F5G3. Full length genomic DNA of candidate genes was amplified by PCR and sequenced to find the mutant site. To confirm the mutated gene, genomic complementation and T-DNA insertion mutant were used.

### Trypan blue staining and DAB staining

Trypan blue and DAB staining was performed as previously described [69,70].

### Pathogen resistance assay

The pathogen resistance assay on *Pseudomonas syringae* pv *tomato* (*P*.*s*.*t*.) strain DC3000 was performed as described previously [32]. Briefly, 3-week-old plants were dipped by the bacterial suspension containing 10 mM MgCl_2_ and 0.025% Silwet L–77. The concentrations of pathogen for virulent strain (*P*.*s*.*t*. DC3000) and avirulent strains (*P*.*s*.*t*. DC3000 with *avrRmp1*, *avrRpt2* and *avrRps4*) are OD_600_ of 0.05 and 0.2, respectively. The dipped leaves were collected at 2 hour and 4 day post-inoculation. Three replicate samples were collected to measure the resistance to pathogen.

### qRT-PCR analysis

Total RNA isolated from 2-week-old plants grown at 22°C or 3-week-old plants grown at 16°C using TRIzol reagent (Invitrogen). The quantitative real-time PCR was performed using a SYBR Green PCR Master Mix kit (Takara). The relative expression levels were calculated as described (Huang et al., 2010). Gene-specific primers were listed in S1 Table.

### Plasmid construction and plant transformation

To obtain the *IBR5*::*IBR5* construct, the genome fragment of *IBR5* (from ~2kb upstream of ATG to stop codon) was amplified and cloned into pCAMBIA1300 vector. To generate *Super*:*IBR5* and *Super*:*SGT1b* constructs, cDNAs of *IBR5* and *SGT1b* were amplified and cloned into the pSuper1300-Myc/GFP/Flag vectors containing a Super promoter.

To generate *Super*:*CHS3-1-Myc*, *Super*:*SNC1-1-Myc*, *Super*:*RPM1-Myc* and *Super*:*RPS4-Myc*, genomic DNA of *chs3-1*, *snc1-1*, *RPM1* and *RPS4* were amplified and cloned into the pSuper1300-Myc vectors containing a Super promoter. The primers were listed in S1 Table.

*Agrobacterium tumefaciens* stain of GV3101 carrying different constructs was transformed into wild-type or mutant plants via standard floral dipping method [71].

### Yeast two-hybrid assay

To create bait and prey plasmids, fragments were amplified by different primers and cloned into pGBKT7 and pGADT7. All primer sequences are listed in S1 Table. Bait and prey plasmids were co-transformed into yeast strain AH109. Yeast strains containing the bait and prey plasmids were cultured in SD-Trp-Leu liquid medium to OD_600_ = 0.4–0.6. Interaction was tested on SD-Trp-Leu-His-Ade medium.

### LCI assay

cDNAs of *HSP90* and *SGT1b* were amplified and cloned into nLuc vector. *IBR5* cDNA was amplified and cloned into cLuc vector. *A*. *tumefaciens* strain GV3101 containing different constructs was infiltrated into *N*. *benthamiana* leaves according to the protocol described previously [72]. Firefly Luciferase Complementation Imaging (LCI) assay was performed as described [73].

### Thermal aggregation assay

To clone the *IBR5-His* construct, *IBR5* cDNA was amplified and cloned into pQE80L vector. *HSP90* fragment was amplified and cloned into pMalC2 vector. The thermal aggregation assay was performed as described [74] with some modifications. Citrate synthase from porcine heart (Sigma-Aldrich) was used as substrate. Light scattering was measured by a thermostatically controlled fluorescence spectrophotometer (F–7000). Proteins were diluted by 40 mM HEPES/KOH (pH 7.5) and the reaction temperature was kept at 43°C.

### Coimmunoprecipitation

The plasmids were purified and transformed into *Arabidopsis* mesophyll protoplasts [75] or *N*. *benthamiana* leaves [72]. Proteins were extracted with extracting buffer containing 150 mM NaCl, 10 mM Tris-HCl pH 7.6, 2 mM EDTA, 0.5% NP–40 and 1 x protease inhibitor cocktail (Roche). The protein extracts were then incubated with anti-Myc beads (Sigma-Aldrich) at 4°C for 2 hour. After washing five times with extraction buffer, the samples were used for immunoblotting with anti-HA (Sigma-Aldrich) or anti-Flag (Sigma-Aldrich) antibodies.

## Supporting Information

S1 Fig The responses of *ibr5* mutants to auxin. Auxin responses of wild type Col, *chs3-1*, *ibr5-3*, *ibr5-7*, and *chs3-1 ibr5-7*. Four-day-old seedlings grown on MS medium with 10 μM IBA, 10 nM IAA, or 50 nM 2,4-D. The relative root length was calculated as root length of seedling grown with hormones versus control (without hormones).
(TIF)

S2 Fig Subcellular localization of IBR5 and CHS3 proteins. (A) Subcellular localization of IBR5 in *Arabidopsis* protoplasts. The *Super*:*IBR5-GFP* and *Super*:*GFP* plasmids were transformed into *Arabidopsis* protoplasts, and the signals were detected using a confocal laser-scanning microscope. The green fluorescence signals, chlorophyll red autofluorescence, an overlay of the green and red signals, and bright-field images are shown. Bars: 20 μm. (B) The subcellular localization of IBR5 and CHS3 proteins in the roots of *Super*:*IBR5-GFP* and *CHS3*:*chs3-2D-GFP* transgenic plants. Bar: 100 μm.
(TIF)

S3 Fig Inteaction of SGT1b and CHS3 in plant. (A) Inteaction of SGT1b and TIR domain of CHS3 in *vivo*. *Super*:*CHS3-TIR-Myc* or *Super*:*Myc* was co-expressed with *Super*:*SGT1b-Flag* in *Arabidopsis* protoplasts. An anti-Myc antibody was used for immunprecipitation and the immunprecipitated proteins were analyzed by immunoblotting using an anti-FLAG antibody. (B) Interaction of SGT1b and full-length CHS3 *in vivo*. *Super*:*SGT1b-Flag* and *Super*:*CHS3-1-Myc* or *Super*:*Myc* was co-expressed with *Super*:*SGT1b-Flag* in *N*. *benthamiana* leaves. An anti-Myc antibody was used for immunprecipitation and the immunprecipitated proteins were analyzed by immunoblotting using an anti-FLAG antibody.
(TIF)

S4 Fig Alignment of TIR domains of CHS3, RPP4 and SNC1 proteins. (TIF)

S5 Fig Phenotypes of *chs1 ibr5* and *chs2 ibr5* mutants. (A) Morphology (top panel) and trypan blue (bottom panel) staining of 4-week-old Col, *ibr5-3*, *chs1-2*, and *chs1 ibr5*. Ten-day-old seedlings grown at 22°C were transferred to 16°C for additional 3 weeks. Bar: 200 μm. (B) The expression of *PR* genes in plants in (A). The error bars represent the SD of three replicates. (C) Morphology (top panel) and trypan blue (bottom panel) staining of Col, *ibr5-7*, *chs2-1* and *chs2 ibr5*. Two-week-old seedlings grown at 22°C were transferred to 4°C for an additional week. Bar: 200 μm. (D) Expression of *PR* genes in plants in (C). The error bars represent the SD of three replicates.
(TIF)

S6 Fig Quantification of *Hyaloperonospora arabidopsidis* (*H*.*a*.) Emwa1 sporulation on wild type, *ibr5-3*, *ibr5-7* and *rpp4-r26* plants. 2-week-old seedlings were sprayed with *H*.*a*. Emwa1 at a concentration of 200,000 spores per 1 mL of water. The spores were quantified 7 day after inoculation. The data are mean values of five replicates ± SD. The experiment was repeated three times with similar results.
(TIF)

S7 Fig Interaction of IBR5 with RPS1 and RPM1. (A, B) Interaction of IBR5 and RPS4, RPM1 (A) and RRS1 (B) in yeast. TIR domains of RPS4, RPM1 (A), CHS3 and RRS1 (B) were fused with the pGADT7 vector. IBR5 and mutated IBR5^C129S^ were fused with the pGBKT7 vector. The constructs were transformed into AH109 yeast cells and spotted onto SD media lacking Trp and Leu (-2SD) or lacking Trp, Leu, His and Ade (-4SD). The experiments were performed three times with similar results. (A, B) Interaction of IBR5 with TIR domains of RPS4, RPM1 (A), CHS3 and RRS1 (B) in yeast. The experiments were performed three times with similar results. (C) Interaction of IBR5 and full-length RPS4 *in vivo*. Total proteins were extracted from *N*. *benthamiana* leaves transfected with *35S*: *HF-IBR5* and *Super*:*RPS4-Myc* or *Super*:*Myc*, and were immunoprecipated with anti-Myc antibody. The proteins from crude lysates (Input) and the immunoprecipated proteins were detected using an anti-HA antibody. (D) Interaction of IBR5 and full-length RPM1 *in vivo*. Total proteins were extracted from *N*. *benthamiana* leaves transfected with *35S*:*HF-IBR5* and *Super*:*RPM1-Myc* or *Super*:*Myc*, and were immunoprecipated with anti-Myc antibody. The proteins from crude lysates (Input) and the immunoprecipated proteins were detected using an anti-HA antibody. (E) The effect of IBR5 on RPM1 protein level in *Arabidopsis* protoplasts. *Super*:*RPM1-Myc* and *35S*:*HF-IBR5* or *35S*:*HF* constructs were co-expressed in *Arabidopsis* protoplasts. IBR5 was detected with anti-HA antibody and RPM1 was detected using an anti-Myc antibody. The *GFP-Myc* construct was used as a control for transformation efficiency.
(TIF)

S8 Fig *chs3 ibr5* phenotype is not rescued by *mpk12* mutant. (A) Diagram of genomic fragment of the *MPK12* gene. The exons are presented as dark blue boxes, UTR regions are presented as light blue boxes, and intron regions are presented as lines. The position of *mpk12-3* (SAIL_543_F07) is shown. (B) RT-PCR analysis of *MPK12* in Col and *mpk12*. The *EF1α* was used as a control. (C) Responses of *MPK12-RNAi* and *mpk12-3* mutants to auxin. (D) Phenotypes of Col, *chs3-1*, *ibr5-7*, *chs3 ibr5*, *mpk12*, *chs3 mpk12* and *chs3 ibr5 mpk12* grown in soil at 16°C for 3 weeks.
(TIF)

S1 Table Gene-specific primers used in this study. (DOC)
