# Correction: IBR5 Modulates Temperature-Dependent, R Protein CHS3-Mediated Defense Responses in *Arabidopsis*

**DOI:** 10.1371/journal.pgen.1005795

**Published:** 2016-01-06

**Authors:** The PLOS Genetics Staff

The affiliation for the seventh author is incorrect. Shuhua Yang is not affiliated with #2, but with #1 State Key Laboratory of Plant Physiology and Biochemistry, College of Biological Sciences, National Plant Gene Research Center, China Agricultural University, Beijing, China. The publisher apologizes for the error.
