# Supplementary figures and images for: IBR5 Modulates Temperature-Dependent, R Protein CHS3-Mediated Defense Responses in Arabidopsis
Source: PLoS Genet. 2015 Oct 9;11(10):e1005584. doi: 10.1371/journal.pgen.1005584 (PMC4599859; doi:10.1371/journal.pgen.1005584)

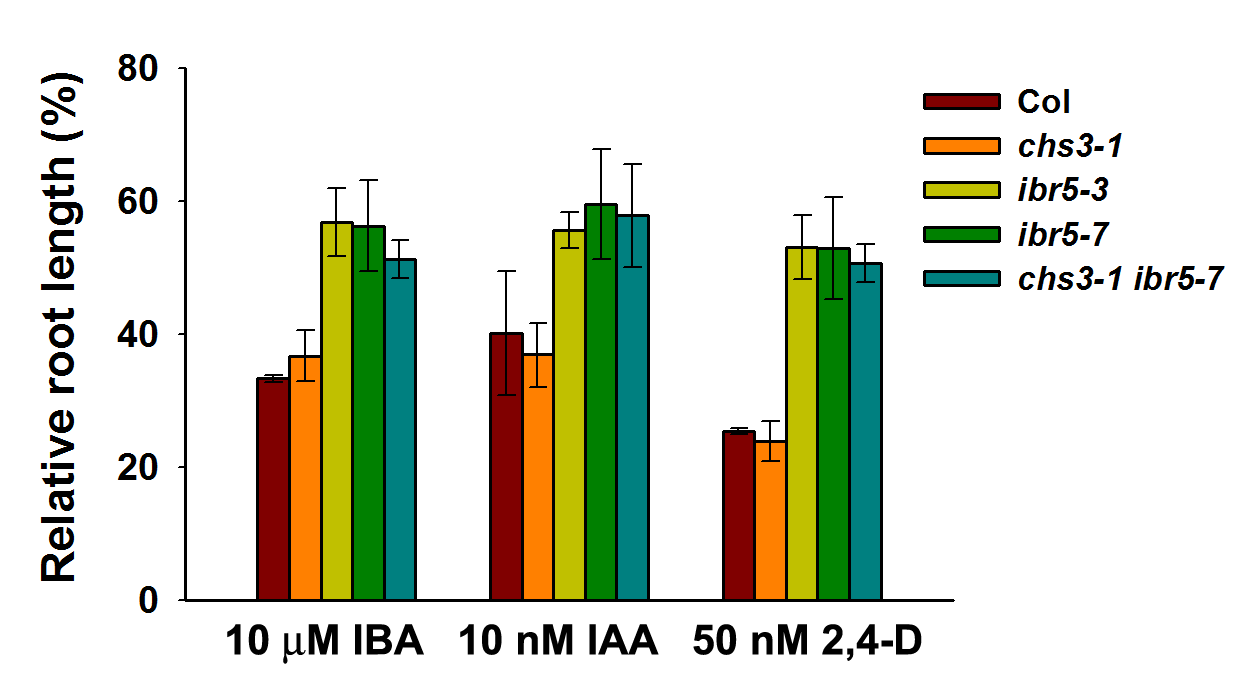

Supplement: S1 Fig — Auxin responses of wild type Col, chs3-1, ibr5-3, ibr5-7, and chs3-1 ibr5-7. Four-day-old seedlings grown on MS medium with 10 μM IBA, 10 nM IAA, or 50 nM 2,4-D. The relative root length was calculated as root length of seedling grown with hormones versus control (without hormones). (TIF) [file pgen.1005584.s001.tif]

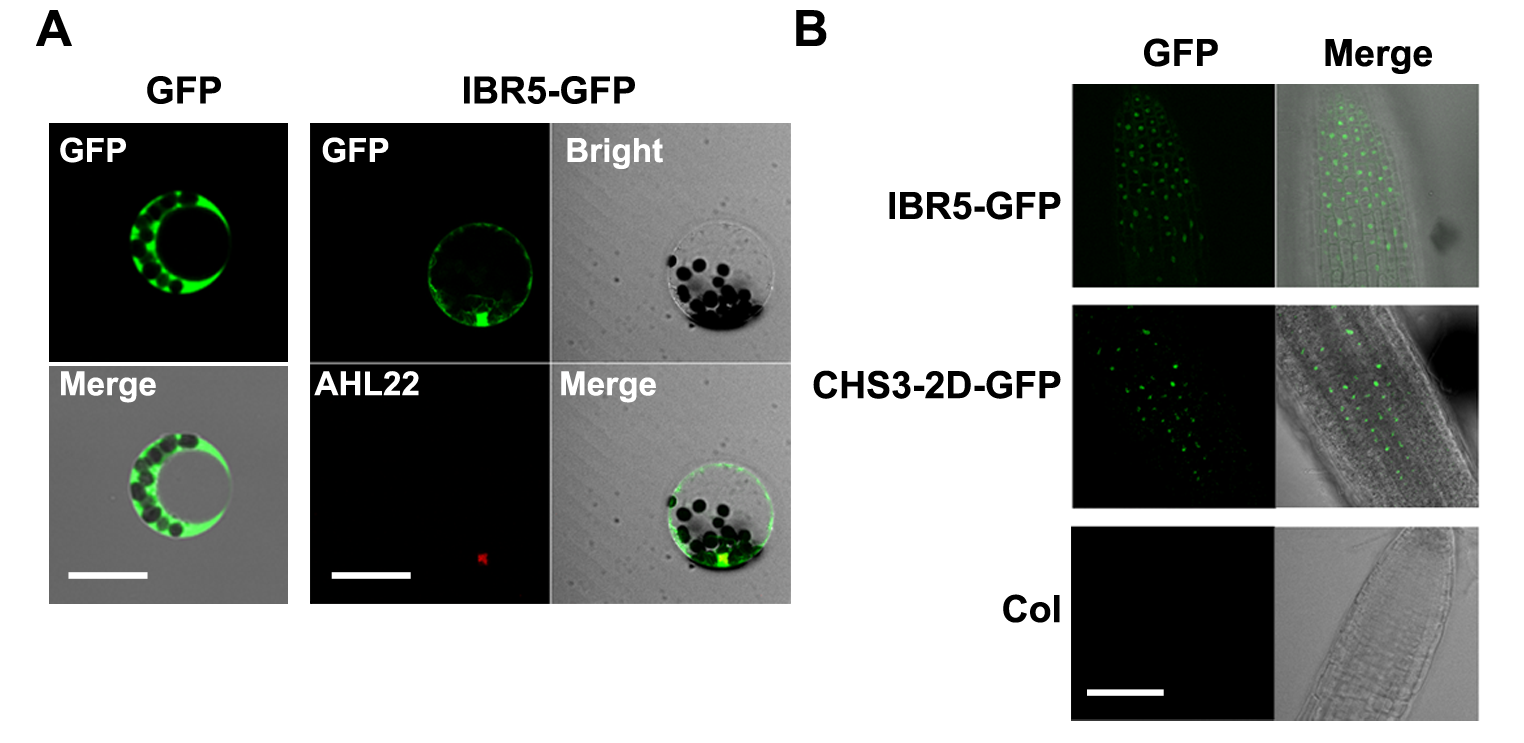

Supplement: S2 Fig — (A) Subcellular localization of IBR5 in Arabidopsis protoplasts. The Super:IBR5-GFP and Super:GFP plasmids were transformed into Arabidopsis protoplasts, and the signals were detected using a confocal laser-scanning microscope. The green fluorescence signals, chlorophyll red autofluorescence, an overlay of the green and red signals, and bright-field images are shown. Bars: 20 μm. (B) The subcellular localization of IBR5 and CHS3 proteins in the roots of Super:IBR5-GFP and CHS3:chs3-2D-GFP transgenic plants. Bar: 100 μm. (TIF) [file pgen.1005584.s002.tif]

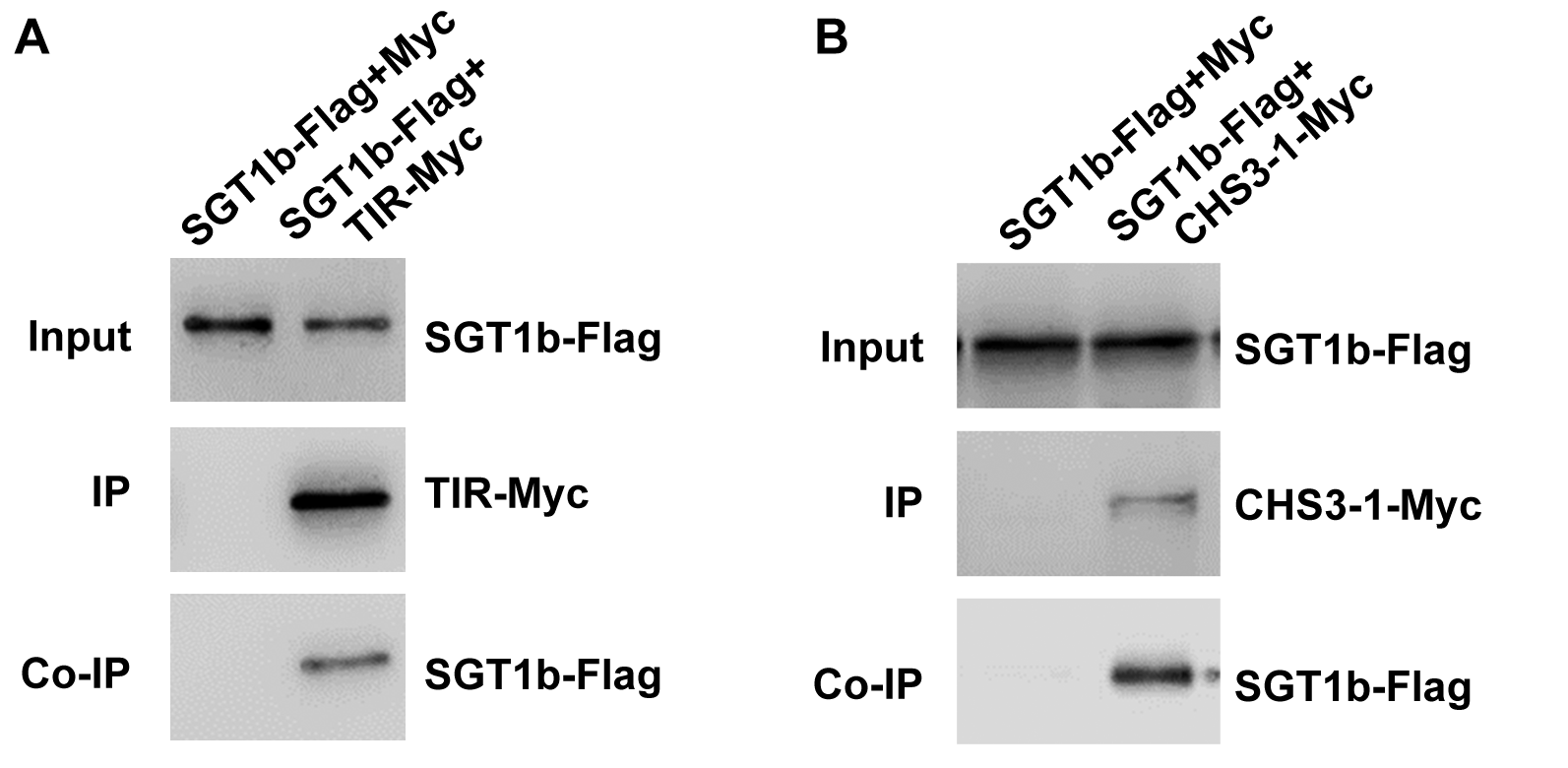

Supplement: S3 Fig — (A) Inteaction of SGT1b and TIR domain of CHS3 in vivo. Super:CHS3-TIR-Myc or Super:Myc was co-expressed with Super:SGT1b-Flag in Arabidopsis protoplasts. An anti-Myc antibody was used for immunprecipitation and the immunprecipitated proteins were analyzed by immunoblotting using an anti-FLAG antibody. (B) Interaction of SGT1b and full-length CHS3 in vivo. Super:SGT1b-Flag and Super:CHS3-1-Myc or Super:Myc was co-expressed with Super:SGT1b-Flag in N. benthamiana leaves. An anti-Myc antibody was used for immunprecipitation and the immunprecipitated proteins were analyzed by immunoblotting using an anti-FLAG antibody. (TIF) [file pgen.1005584.s003.tif]

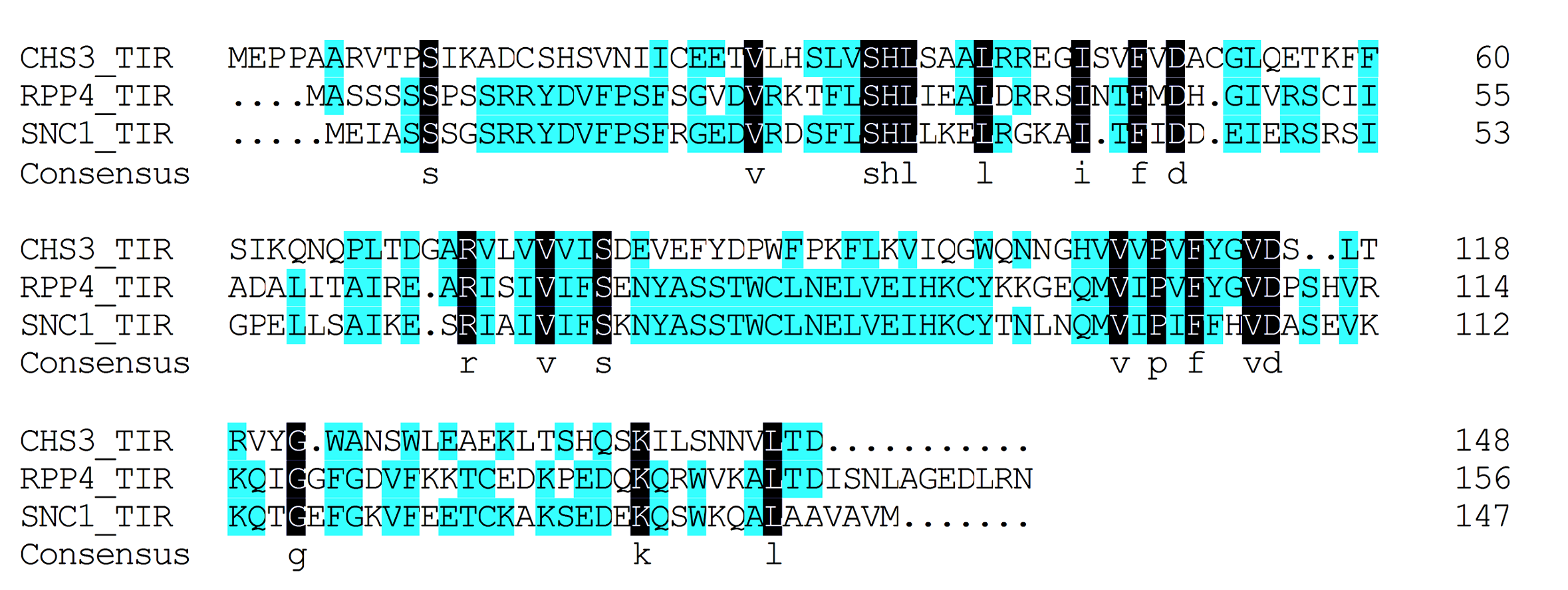

Supplement: S4 Fig — (TIF) [file pgen.1005584.s004.tif]

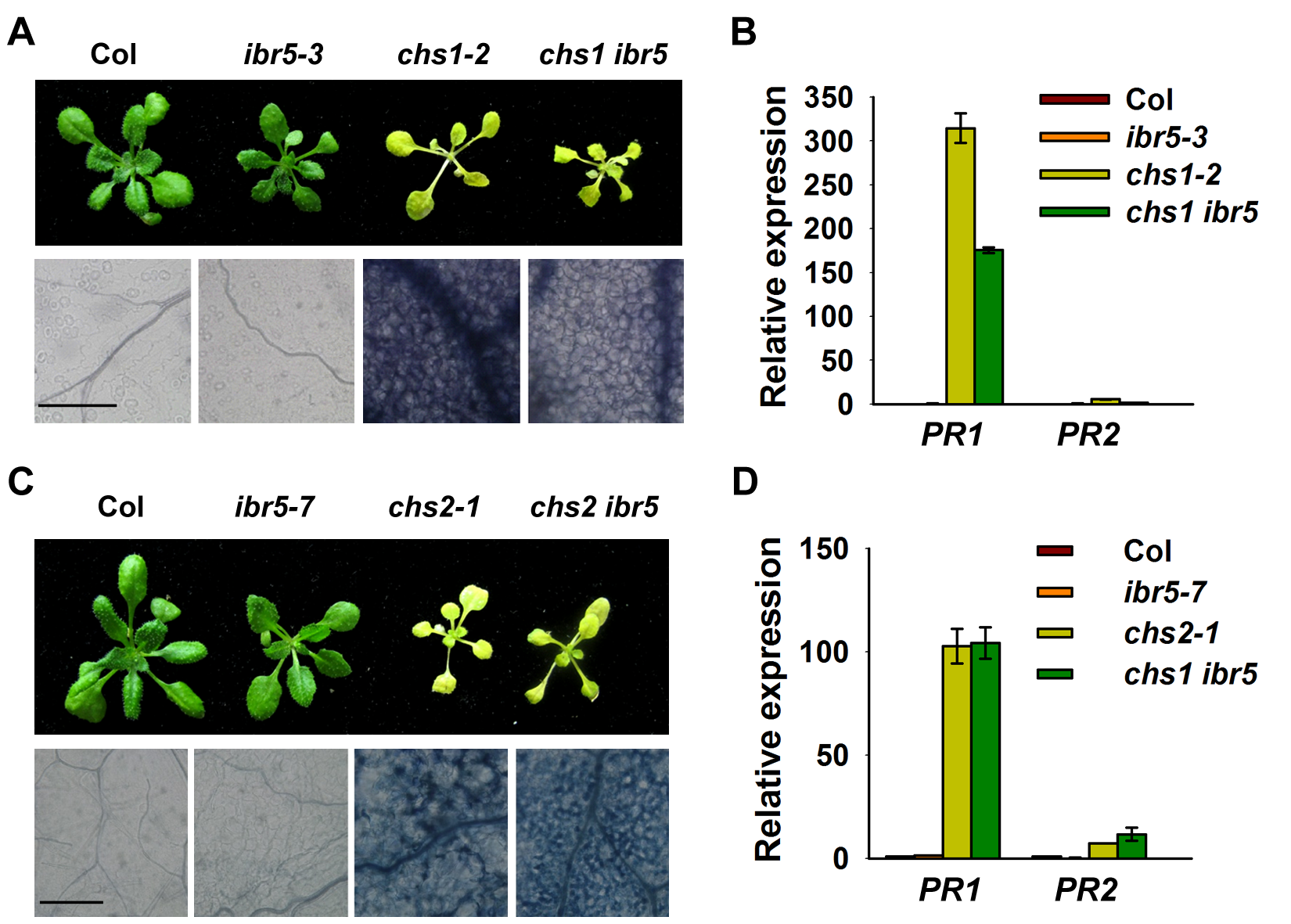

Supplement: S5 Fig — (A) Morphology (top panel) and trypan blue (bottom panel) staining of 4-week-old Col, ibr5-3, chs1-2, and chs1 ibr5. Ten-day-old seedlings grown at 22°C were transferred to 16°C for additional 3 weeks. Bar: 200 μm. (B) The expression of PR genes in plants in (A). The error bars represent the SD of three replicates. (C) Morphology (top panel) and trypan blue (bottom panel) staining of Col, ibr5-7, chs2-1 and chs2 ibr5. Two-week-old seedlings grown at 22°C were transferred to 4°C for an additional week. Bar: 200 μm. (D) Expression of PR genes in plants in (C). The error bars represent the SD of three replicates. (TIF) [file pgen.1005584.s005.tif]

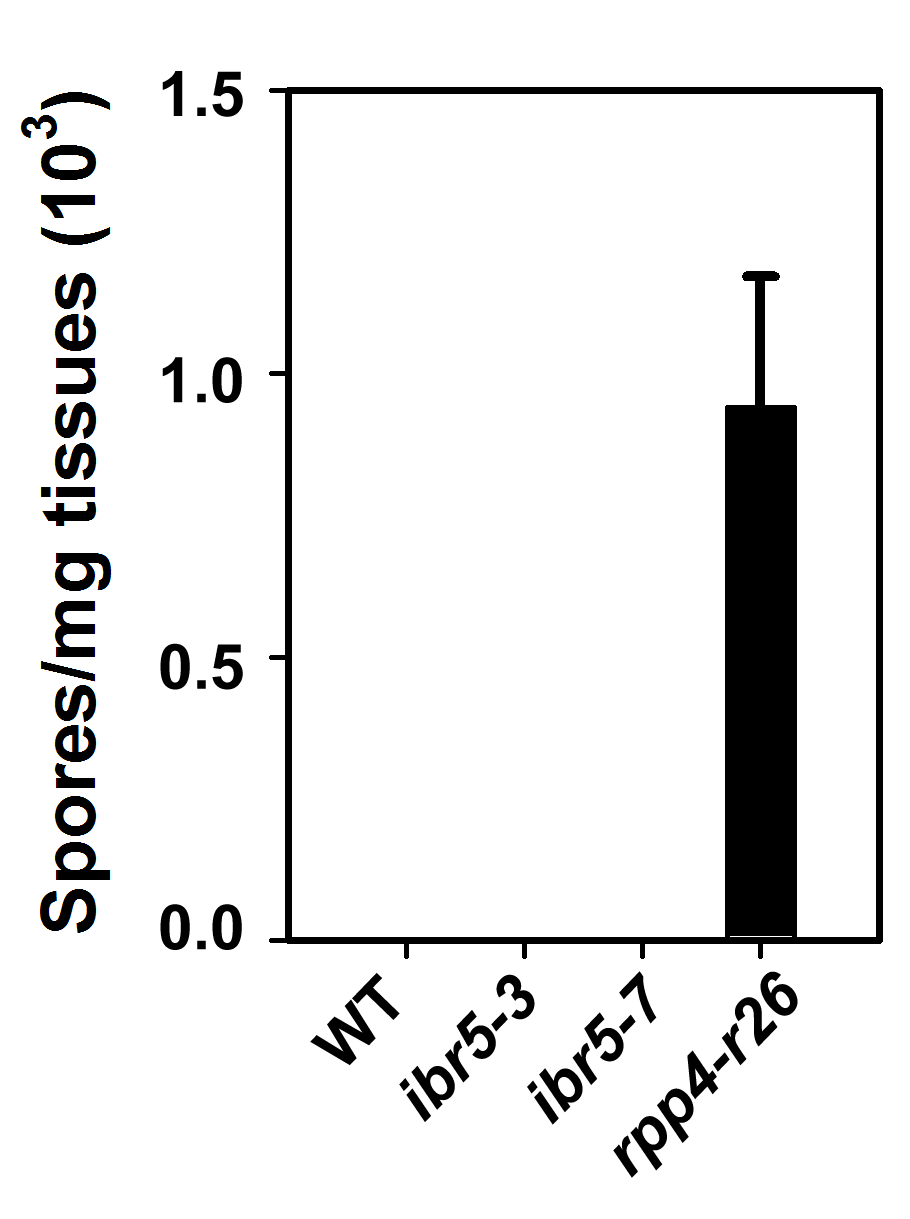

Supplement: S6 Fig — 2-week-old seedlings were sprayed with H.a. Emwa1 at a concentration of 200,000 spores per 1 mL of water. The spores were quantified 7 day after inoculation. The data are mean values of five replicates ± SD. The experiment was repeated three times with similar results. (TIF) [file pgen.1005584.s006.tif]

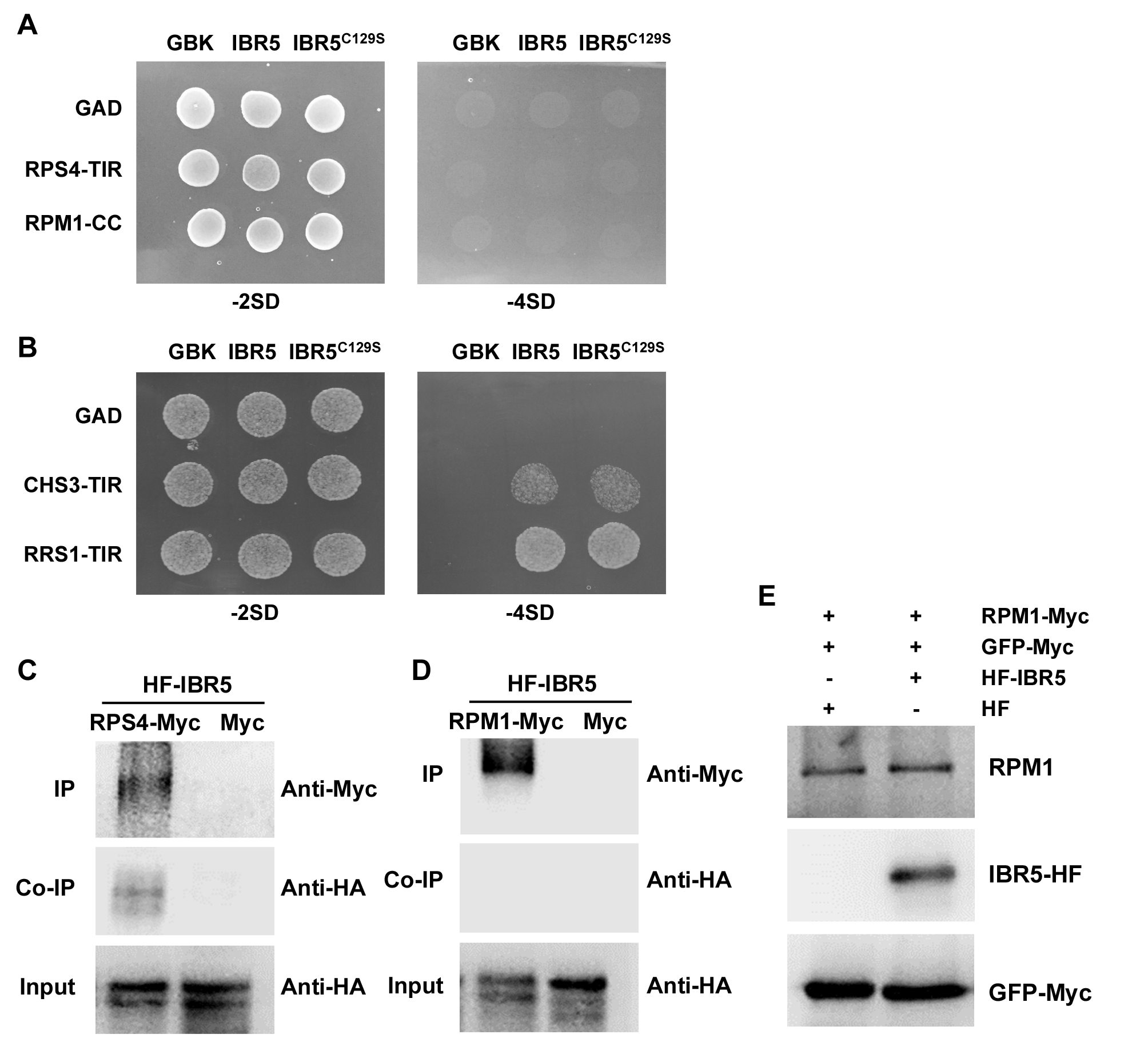

Supplement: S7 Fig — (A, B) Interaction of IBR5 and RPS4, RPM1 (A) and RRS1 (B) in yeast. TIR domains of RPS4, RPM1 (A), CHS3 and RRS1 (B) were fused with the pGADT7 vector. IBR5 and mutated IBR5C129S were fused with the pGBKT7 vector. The constructs were transformed into AH109 yeast cells and spotted onto SD media lacking Trp and Leu (-2SD) or lacking Trp, Leu, His and Ade (-4SD). The experiments were performed three times with similar results. (A, B) Interaction of IBR5 with TIR domains of RPS4, RPM1 (A), CHS3 and RRS1 (B) in yeast. The experiments were performed three times with similar results. (C) Interaction of IBR5 and full-length RPS4 in vivo. Total proteins were extracted from N. benthamiana leaves transfected with 35S: HF-IBR5 and Super:RPS4-Myc or Super:Myc, and were immunoprecipated with anti-Myc antibody. The proteins from crude lysates (Input) and the immunoprecipated proteins were detected using an anti-HA antibody. (D) Interaction of IBR5 and full-length RPM1 in vivo. Total proteins were extracted from N. benthamiana leaves transfected with 35S:HF-IBR5 and Super:RPM1-Myc or Super:Myc, and were immunoprecipated with anti-Myc antibody. The proteins from crude lysates (Input) and the immunoprecipated proteins were detected using an anti-HA antibody. (E) The effect of IBR5 on RPM1 protein level in Arabidopsis protoplasts. Super:RPM1-Myc and 35S:HF-IBR5 or 35S:HF constructs were co-expressed in Arabidopsis protoplasts. IBR5 was detected with anti-HA antibody and RPM1 was detected using an anti-Myc antibody. The GFP-Myc construct was used as a control for transformation efficiency. (TIF) [file pgen.1005584.s007.tif]

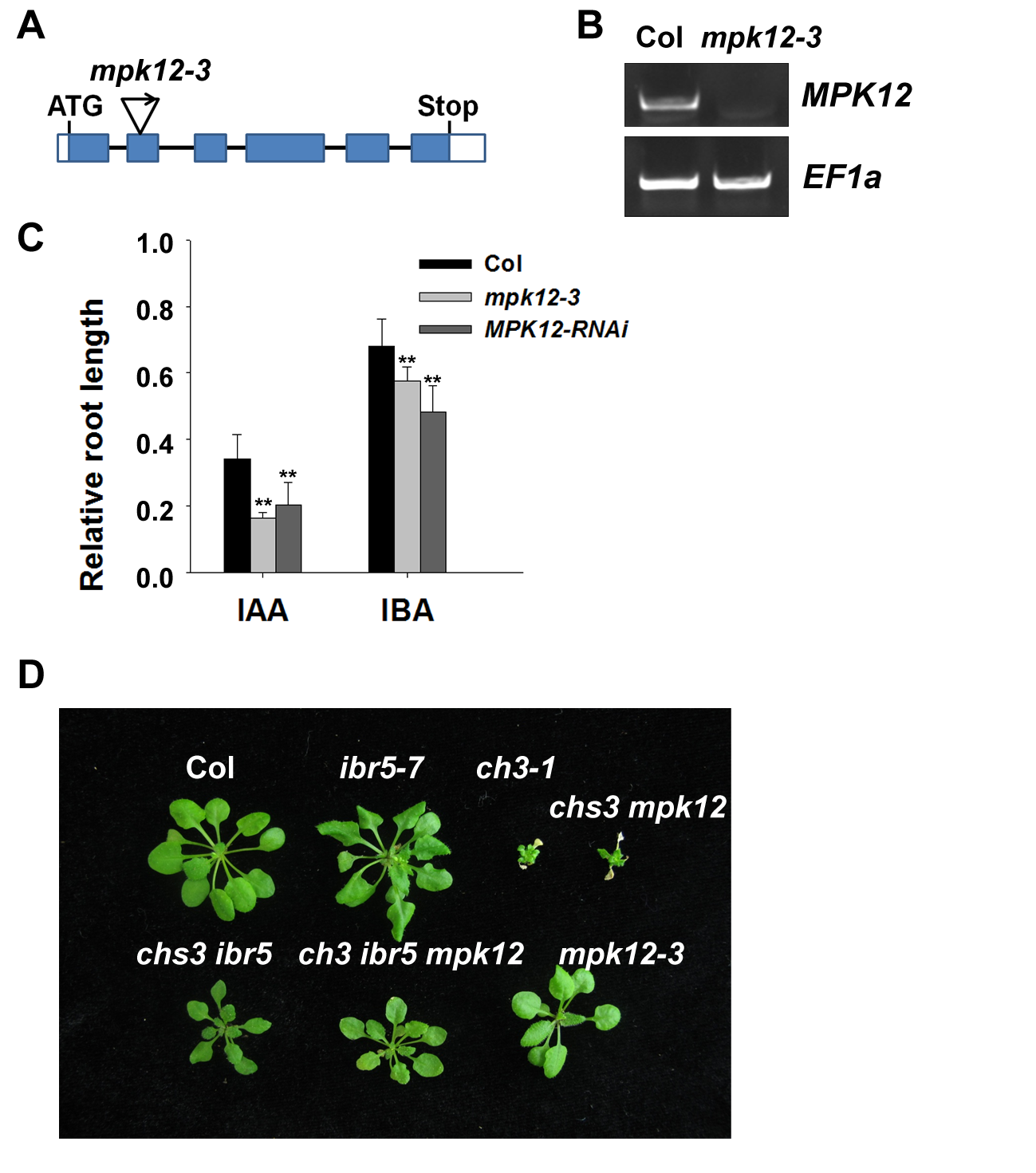

Supplement: S8 Fig — (A) Diagram of genomic fragment of the MPK12 gene. The exons are presented as dark blue boxes, UTR regions are presented as light blue boxes, and intron regions are presented as lines. The position of mpk12-3 (SAIL_543_F07) is shown. (B) RT-PCR analysis of MPK12 in Col and mpk12. The EF1α was used as a control. (C) Responses of MPK12-RNAi and mpk12-3 mutants to auxin. (D) Phenotypes of Col, chs3-1, ibr5-7, chs3 ibr5, mpk12, chs3 mpk12 and chs3 ibr5 mpk12 grown in soil at 16°C for 3 weeks. (TIF) [file pgen.1005584.s008.tif]
