# Supplementary material for: IBR5 Modulates Temperature-Dependent, R Protein CHS3-Mediated Defense Responses in Arabidopsis
Source: PLoS Genet. 2015 Oct 9;11(10):e1005584. doi: 10.1371/journal.pgen.1005584 (PMC4599859; doi:10.1371/journal.pgen.1005584)
Supplement: S1 Table — (DOC) [file pgen.1005584.s009.doc]

**Table S1. Gene-specific primers used in this study.**

| **For cloning and complementation** | |
| --- | --- |
| IBR5-F | atgaggaagagagaaagagag |
| IBR5-R | GGTACCAGAGCCATCCATTGCAATATCAC |
| IBR5-com-F | CATTCAAGGGCTTAGTTGAGAGTGT |
| IBR5-com-R | GTCGACCTAAGAGCCATCCATTGCAATATCAC |
| SGT1B-1 | ATGGCCAAGGAATTAGCAGAGAAAG |
| SGT1B-2 (KpnI) | GGTACCATACTCCCACTTCTTGAGCTCCATG |
| SGT1B-KpnI-F | GGTACCATGGCCAAGGAATTAGCAGAG |
| SGT1B-BglII-R | AGATCTATACTCCCACTTCTTGAGCTC |
| HSP90-F | ATGGCGGACGCAGAAACCTTTG |
| HSP90-R | GTCAACTTCCTCCATCTTGCTAC |
| CHS3-F- SpeI | ACATTTAAATACTAGTATGGAACCACCAGCTG |
| CHS3-R-SpeI | TACCGGATCCACTAGTGACACTGATCCCCCTG |
| SNC1-1-F-SpeI | ACATTTAAATACTAGTATGGAGATAGCTTCTTCTTCTG |
| SNC1-1-R-SpeI | TACCGGATCCACTAGTGTTACCAGAAACAGGAAACAAG |
| SalI-RPM1-F | GTCGACatggcttcggctactgttg |
| KpnI-RPM1-R | GGTACCagatgagaggctcacatag |
| RPS4-F-SpeI | ACATTTAAATACTAGTATGGAGACATCATCTATTTCCAC |
| RPS4-R-SpeI | TACCGGATCCACTAGTGAAATTCTTAACCGTGTGCATGATC |
| NdeI-RRS1T-F | GGAATTCcatatgaccaattgtgaaaag |
| BamHI-RRS1T-R | cgggatcctcatccaattcgtccaac |
| NdeI-RPS4T-F | GGAATTCcatatggagacatcatctat |
| BamHI-RPS4T-R | cgggatcctcatattccggtcaacgctg |
| NdeI-RPM1C-F | GGAATTCcatatggcttcggctactgt |
| ClaI-RPM1C-R | ccatcgatctaccctacaagacta |
| EcoRI-TIR-F (CHS3) | GTGAATTCatggaaccaccagctgctcgtg |
| BamHI-TIR-R (CHS3) | gggatcctgagtccgtgagtacattattgc |
| NdeI-TIR-F (CHS2) | TcatatgGCTTCTTCTTCTTCTTCTC |
| BamHI-TIR-R (CHS2) | gggatccTCAATCTTCAATTCCG |
| NdeI-TIR-F (SNC1) | Tcatatggagatagcttcttcttc |
| BamHI-TIR-R (SNC1) | TGGATCCTCAgactaagtcgccaaa |
| NdeI-IBR5-F | gcatatgatgaggaagagagaaagaga |
| NdeI-SGT1b-F | gcatatgatggccaaggaattagcagag |
| SGT1b-EcoRI-R | CGAATTCTCAATACTCCCACTTCTTGAG |
| NdeI-HSP90-F | gcatatgatggcggacgcagaaacctttg |
| HSP90-BamHI-R | GGGATCCttagtcaacttcctccatcttg |
| **For genotyping** | |
| IBR5-P1 | CCTATGTGCCAGAATCTCTACAG |
| IBR5-P2  LB1 | ggatccctaagagccatccattgcaatatc  gctgttgcccgtctcactggtg |
| MPK12-F | gactctgcttcttgttaccatac |
| MPK12-R | AGGAActgtaacaggaagacaag |
| GFP-P1 | CTGCAGatggtgagcaagggcgaggagc |
| GFP-P2 | GGTACCTTACTTGTACAGCTCGTCCATGCCGAG |
| **For Real-Time PCR analysis** | |
| IBR5-1 | GAACACGAGAGATTCCTTGAGTC |
| IBR5-2 | GGATCCATGAGGAAGAGAGAAAGAGAG |
| PR1-1 | CACATCCGAGTCTCACTGAC |
| PR1-2 | CAGACTCATACACTCTGGTG |
| PR2-1 | AAGGAGCTTAGCCTCACCAC |
| PR2-2 | CACAACGTCCGATGGACTTG |
| ACTIN2-1 | GGTAACATTGTGCTCAGTGGTGG |
| ACTIN2-2 | AACGACCTTAATCTTCATGCTGC |
| SNC1-F | ATATGGAGATAGCTTCTTCTTCTG |
| SNC1-R  CHS3-F | AAGATCATATCCGGCCATAACTG  AGAGGACACCAGAGAGTTACA |
| CHS3-R | CTTTCGTCTGCGTGCTTTATTT |
| RPM1-F | CCAAGCAGCATTATTACCGCC |
| RPM1-R | cgctgagagtgtagttttccc |
| RPS4-F | CCTAACATTATGGGCATCATCA |
| RPS4-R | CCGCCTTCACAATTTCATTGA |
| RRS1-F | CGTGTGCATCAGCTGCGTAGAAG |
| RRS1-R | GAACCTTGGCGAACTTGTCAAGC |
| RPP4-F | GACAATAATCCCACCATAGCCTTT |
| RPP4-R | GAAGGCACTCAAGGCCTCATTTAC |
